# Population Pharmacokinetics and Exposure–Response Analysis of Oral Pixavir Marboxil in Adults and Adolescents with Influenza

**DOI:** 10.3390/pharmaceutics18050550

**Published:** 2026-04-30

**Authors:** Ying Yang, Chenyu Wang, Xiaoqin Liu, Peijin Li, Chong Su, Zheng Jiao

**Affiliations:** 1 Joincare Pharmaceutical Group Industry Co., Ltd., Shenzhen 518057, China; yangying@joincare.com (Y.Y.); suchong@joincare.com (C.S.); 2 Shanghai Chest Hospital, Shanghai Jiao Tong University School of Medicine, Shanghai 200030, China; c.wang@lacdr.leidenuniv.nl (C.W.); 18111030003@fudan.edu.cn (X.L.)

**Keywords:** pixavir marboxil, population pharmacokinetics, exposure–response relationship, influenza, adult, adolescents

## Abstract

**Background/Objectives**: Pixavir marboxil is a novel oral cap-dependent endonuclease inhibitor that is rapidly converted to its active metabolite pixavir. This study aimed to characterize the population pharmacokinetics (PopPK) of pixavir, identify clinical covariates affecting its pharmacokinetics, and evaluate the exposure–response relationship to inform optimal dosing in the treatment of acute influenza. **Methods**: Data were pooled from three clinical studies spanning Phase I to Phase III trials and included adults and adolescents aged ≥12 years. PopPK modeling was conducted using NONMEM software. Individual exposure metrics, including the 24-h post-dose concentration, maximum concentration, and area under the concentration-time curve from time zero to infinity, were derived using Bayesian estimation based on the final PopPK model. Efficacy endpoints included time to alleviation of influenza-related symptoms and changes in viral titer and viral RNA load from baseline to Day 2. Safety was evaluated through the incidence of treatment-related adverse events. **Results**: Pixavir pharmacokinetics were best described by a two-compartment model. Body weight significantly influenced clearance and central volume of distribution, while dose amount had a notable effect on bioavailability. Food, sex and age had no clinically meaningful impact on pharmacokinetics. Exposure–response analysis showed no clear relationship between pixavir exposure and both efficacy and safety endpoints within the studied dosing range. **Conclusions**: The weight-based dosing regimen of pixavir marboxil (40 mg for <80 kg and 80 mg for ≥80 kg) is supported, as it reduces body-weight-driven exposure variability and maintains exposure within the clinically characterized range in adults and adolescents with acute influenza.

## 1. Introduction

Pixavir marboxil (product code TG-1000) is a novel cap-dependent endonuclease inhibitor for the treatment of influenza. After oral administration, pixavir marboxil is rapidly hydrolyzed by intestinal carboxylesterases to its active metabolite, pixavir (product code TG-0527), which has potent antiviral activity against both influenza A and influenza B viruses [1,2]. Recent clinical studies have demonstrated that a single oral dose of pixavir marboxil is effective for the treatment of uncomplicated influenza A and influenza B in adolescents aged 12 years and older and in adults, leading to its approval by the National Medical Products Administration of China [1].

After oral administration, plasma concentrations of the prodrug pixavir marboxil are generally below the lower limit of quantification (LLOQ) because of its rapid conversion to pixavir, the circulating active moiety. Early clinical pharmacology studies showed that pixavir reaches its maximum concentrations (C_max_) at approximately 3.5 h after dosing and then declines in a biphasic manner. Exposure increased less than proportionally with dose across the investigated dose range, suggesting complexity in absorption and/or presystemic disposition. Food also affects pixavir pharmacokinetics: administration with a high-fat meal reduces the C_max_ and area under the concentration-time curve from time zero to infinity (AUC_inf_) by approximately 41% and 19%, respectively, and delays the time to reach C_max_ by about 1.5 h [1,3]. In adult patients with influenza, the apparent volume of distribution after a single 40 mg oral dose is approximately 614 L. Pixavir is highly plasma protein-bound (>96%), undergoes UGT1A3-mediated glucuronidation as well as oxidative metabolism, and is eliminated predominantly via feces. The terminal half-life of pixavir is approximately 36 h [1].

Several clinical studies of pixavir marboxil have been completed, including first-in-human single-dose escalation and food-effect studies in healthy volunteers [3], followed by Phase II and Phase III efficacy trials in patients with acute uncomplicated influenza. These studies have provided descriptive pharmacokinetic (PK) information (e.g., concentration–time profiles and noncompartmental exposure summaries) and basic clinical pharmacology findings such as food effects and dose–exposure patterns.

However, an important gap remains between descriptive PK findings and model-informed dose optimization. An integrated population pharmacokinetic (PopPK) and exposure–response (ER) analysis that pools data across phases, quantifies inter-individual variability (IIV), and evaluates exposure–outcome relationships in adolescents and adults is still lacking. Such analyses address this gap by linking heterogeneous PK data with efficacy and safety endpoints [4,5,6]. These integrated approaches offer crucial insights to guide dose optimization and support evidence-based dose selection.

The present study aimed to characterize the PopPK of pixavir in patients with uncomplicated influenza, quantify PK variability across demographic subgroups, including adolescents and adults, and identify clinically relevant covariates influencing drug exposure. Additionally, this study sought to evaluate exposure–response relationships for both antiviral efficacy and safety outcomes to inform dose regimen optimization, thereby maximizing clinical benefit while minimizing potential risks.

## 2. Materials and Methods

### 2.1. Data

#### 2.1.1. Clinical Study

Data were obtained from three clinical studies of pixavir marboxil (Joincare Pharmaceutical Group Industry Co., Ltd., Shenzhen, China): a Phase I single- ascending-dose study with a food-effect component in healthy adults (TG-1000-C-01, NCT04495322, www.clinicaltrials.gov), a Phase II study in adults with acute influenza (TG-1000-C-02, NCT04706468, www.clinicaltrials.gov), and a Phase III study in adults and adolescents aged ≥12 years with acute influenza (TG-1000-C-03, CTR20232723). All studies were conducted in accordance with the principles of the Declaration of Helsinki (version 2000) and Good Clinical Practice (GCP) guidelines (version 2020). Each protocol was approved by the institutional review boards or independent ethics committees at each participating site, and all subjects, or guardians for adolescent participants when applicable, provided written informed consent prior to enrollment. Detailed study information is outlined in Table 1.

Data from all three studies were pooled for the PopPK analysis. The ER analysis included only influenza patients from the Phase II and Phase III studies. In Phase III, 261 participants were enrolled; of these, 220 who had PK observations contributed to the PopPK analysis, while all 261, including 49 adolescents with sparse PK sampling, were included in the ER analysis.

#### 2.1.2. Bioassay

Plasma concentrations of pixavir were measured using fully validated liquid chromatography-tandem mass spectrometry (LC-MS/MS) methods. Samples from the Phase I and Phase II studies were analyzed at Labcorp Drug Development Co., Ltd. (Shanghai, China), with an LLOQ of 2.0 ng/mL, whereas Phase III study samples were analyzed at WuXi AppTec Co., Ltd. (Shanghai, China), with an LLOQ of 1.0 ng/mL. Both analytical methods were validated in accordance with ICH M10 guidelines [7] and demonstrated adequate selectivity, precision (inter-run coefficient of variation ≤ 15%), accuracy (bias within ±15%), and stability under relevant storage conditions.

Infectious titers of influenza A and B viruses in human nasopharyngeal swab specimens were quantified at WuXi AppTec using the 50% tissue culture infectious dose (TCID_50_) assay. The assay’s LLOQ was set at 25 TCID_50_/mL (equivalent to 1.40 log_10_ TCID_50_/mL) for both virus types.

Viral RNA loads of influenza A and B viruses were quantified using the TaqMan quantitative PCR method performed on the ABI-QuantStudio™ 7 Flex Real-Time PCR System at WuXi AppTec Co., Ltd. (Shanghai, China). The LLOQ for viral RNA was 3.45 log_10_ copies/mL for influenza A and 3.19 log_10_ copies/mL for influenza B.

### 2.2. Population Pharmacokinetic Analysis

#### 2.2.1. Population Pharmacokinetic Model Development

The PopPK modeling was conducted using NONMEM (version 7.5; ICON plc), with model development and execution supported by PsN (version 5.3.1) and model management performed using Pirana (version 3.0.0). Data visualization and analysis were completed in R (version 4.4.0) with RStudio (version 2024.04.1). The first-order conditional estimation with interaction (FOCE-I) algorithm was applied throughout the analysis.

Subjects were eligible for the PopPK analysis if they received pixavir marboxil and had at least one post-dose PK observation. Concentrations below the lower limit of quantification (BLQ) were handled using a prespecified rule. When BLQ observations accounted for <5% of total PK samples, they were excluded from analysis, consistent with published recommendations that such a small proportion is unlikely to have a meaningful impact on PopPK model results [8,9]. If the proportion exceeded 5%, likelihood-based methods for censored data (e.g., the M3 method) would be explored in the analysis [8,9].

Structural model development was guided by visual inspection of individual and median observed concentration-time profiles by dose group on both linear and semi-logarithmic scales. On this basis, one-, two-, and three-compartment models with first-order oral absorption, with or without an absorption lag time, were evaluated sequentially. Elimination was initially modeled as first-order from the central compartment, consistent with the known elimination pathways of pixavir, including UGT1A3-mediated glucuronidation and fecal excretion. A Michaelis–Menten elimination model was also evaluated as an alternative structural model to assess potential elimination nonlinearity, although the observed less-than-dose-proportional exposure was considered more suggestive of dose-dependent absorption or bioavailability than of saturable clearance.

Inter-individual variability (IIV) was modeled assuming a log-normal distribution, and potential correlations among IIV terms were also explored. Residual unexplained variability (RUV) was described using additive, proportional, and combined additive-proportional error models. The candidate base models were assessed by diagnostic plots, parameter identifiability, Akaike Information Criterion (AIC)/Bayesian Information Criterion (BIC) and overall model stability.

Covariates assessed included demographic factors (age, sex, body weight and body mass index), hepatic and renal function markers (alanine aminotransferase, total bilirubin, total protein and serum creatinine), food status (fasted, standard diet, or high-fat meal), and disease status (healthy subjects versus influenza patients). Potential covariates were first screened through graphical exploration of individual empirical Bayes estimates versus covariates, together with assessment of clinical plausibility, and were then evaluated using a stepwise procedure (forward inclusion: *p* < 0.05, change in objective function value [ΔOFV] > 3.84; backward elimination: *p* < 0.001, ΔOFV > 10.83).

Continuous covariates were modeled using centered parameterizations normalized to a reference value, typically the population median. For each continuous covariate-parameter relationship, linear, proportional, and allometric power functions were evaluated and selection among candidate functions was based on statistical fit, parameter precision, and biological plausibility. For body weight, an allometric power function was prespecified as the primary candidate based on established size–pharmacokinetic theory [10]. Empirically estimated exponents were retained when they provided a statistically superior fit (ΔOFV > 3.84 versus a fixed exponent) and acceptable parameter precision. This approach reflects the recognized methodological debate regarding the applicability of fixed allometric exponents (0.75 for clearance and 1.0 for volumes) to within-species human PK datasets, in which empirically estimated exponents may better characterize body size-related variability in the studied population [10]. Categorical covariates were modeled using indicator-based proportional terms [9,11].

Model evaluation included standard goodness-of-fit (GOF) diagnostics and visual predictive checks (VPCs) based on 1000 Monte Carlo simulations [12,13]. Nonparametric bootstrap analysis (2000 replicates) was also performed to assess model stability and robustness [9]. In addition, model selection considered precision of parameter estimates, AIC/BIC, biological plausibility, and clinical relevance. Shrinkage for both IIV and RUV components was also evaluated [9].

#### 2.2.2. Model Applications

The final PopPK model was used in Monte Carlo simulations to quantify the effects of clinically relevant covariates on pixavir exposure. Simulations were used to generate concentration–time profiles and derived exposure metrics across predefined covariate scenarios. Covariate effects were summarized graphically to facilitate interpretation of their magnitude and potential clinical relevance.

### 2.3. Exposure–Response Analysis

Exposure–response (ER) analyses were performed using individual exposure metrics derived from the final PopPK model of pixavir. For subjects with PK sampling, *post hoc* Bayesian estimates were used to derive individual exposure metrics; for adolescents without PK data, Bayesian predictions based on population priors were used for exposure prediction. Key exposure parameters, including pixavir plasma concentration at 24 h post-dose (C_24h_), C_max_, and AUC_inf_, were calculated and used to evaluate ER relationships for both efficacy and safety endpoints. Only subjects with both available exposure metrics and corresponding endpoint data were included in the analyses; missing endpoint data were not imputed. All ER analyses were conducted using R (version 4.4.0), and figures were produced using ggplot2 and associated visualization packages.

#### 2.3.1. Exposure–Efficacy Analysis

Efficacy endpoints were derived from the Phase II (TG-1000-C-02) and Phase III (TG-1000-C-03) clinical trials and included both clinical and virological measures. Clinical benefit was assessed as the time to alleviation of influenza-related symptoms, defined as the interval from treatment initiation until all seven influenza-related symptoms were rated by the patient as 0 or 1 for at least 21.5 h (90% of a 24 h period) [2]. Virological activity was evaluated by changes in viral RNA load (log_10_ copies/mL) and viral titer (log_10_ TCID50/mL) from baseline to Day 2 post-dose. Day 2 was selected because it represents the period of maximal initial viral suppression, during which treatment-related differences in viral load are most likely to be detected [2]. To characterize exposure–efficacy relationships, a hierarchical modeling strategy was applied. First, exploratory graphical analyses were performed for each exposure metric (AUC_inf_, C_24h_, C_max_) against each efficacy endpoint. Second, candidate exposure–response models, including linear, log-linear, and Emax models, were evaluated in parallel. Model selection was based on convergence status, parameter identifiability, goodness-of-fit diagnostics, and information criteria (AIC/BIC), together with clinical plausibility. More complex models were not retained if they failed to provide a robust and interpretable improvement in fit or yielded unstable parameter estimates compared with simpler alternatives.

#### 2.3.2. Exposure–Safety Analysis

Safety evaluations were conducted throughout all clinical studies in accordance with Common Terminology Criteria for Adverse Events (CTCAE) Version 5.0 guidelines [14]. These evaluations included monitoring treatment-related adverse events (TRAEs), clinical laboratory parameters, vital signs, and electrocardiograms. Adverse events were systematically recorded from the time of initial dosing through the conclusion of the study follow-up period.

Exposure–safety evaluation was prespecified as a stepwise procedure: (1) exploratory summaries by exposure quantiles and treatment group, followed by (2) formal regression-based ER modeling (e.g., logistic/linear models) only if exploratory analyses indicated a potential treatment- or exposure-related safety signal.

## 3. Results

### 3.1. Population Pharmacokinetic Analysis

#### 3.1.1. Population Pharmacokinetic Model Development

A total of 423 subjects from three clinical studies, contributing 3125 pixavir PK observations, were included in the PopPK analysis. All studies were conducted in China, and all enrolled participants were Chinese. Concentrations below the LLOQ accounted for 4.6% of all PK observations and, according to the prespecified rule (<5%), were excluded from model fitting. Most participants exhibited normal renal and hepatic functions. Demographic characteristics of the enrolled subjects are summarized in Table 2.

Visual inspection of the observed profiles (Supplementary Figure S1) showed a short delay in the rise in concentrations after dosing and a biphasic decline on semi-logarithmic plots, supporting inclusion of an absorption lag time and at least two disposition compartments. A two-compartment model with first-order absorption, an absorption lag time, and first-order elimination from the central compartment provided the best overall description of the data. The model was supported by improved statistical fit, acceptable diagnostic performance, and adequate parameter precision, and it adequately captured the observed profiles across the studied dose range. Incorporation of Michaelis–Menten elimination did not improve model performance and resulted in imprecise estimates of Km and Vmax. No clear dose-related change in terminal slope was evident in the observed data. These findings did not support saturable elimination, and first-order elimination was therefore retained as the most parsimonious description of pixavir pharmacokinetics.

IIV was included on apparent clearance (CL/F), central volume of distribution (Vc/F) and the absorption rate constant (*k*_a_). A significant correlation was observed between IIV on CL/F and Vc/F, and inclusion of a covariance term between these parameters improved model performance. RUV was best described by a proportional error model.

Among the covariates examined, body weight was identified as a statistically and clinically relevant predictor of CL/F and Vc/F and was incorporated into the model using allometric functions centered on the population median. Inclusion of body weight reduced the IIV of CL/F from 33.6% in the base model to 26.6% in the final model and reduced the IIV of Vc/F from 46.8% to 39.1%, corresponding to relative reductions of approximately 20.8% and 16.5%, respectively. This covariate relationship was associated with a marked improvement in model fit (ΔOFV > 100; *p* < 0.001).

The estimated allometric exponents were 0.961 for CL/F (RSE 8.2%; bootstrap 95% CI: 0.805–1.13) and 1.69 for Vc/F (RSE 8.5%; bootstrap 95% CI: 1.43–1.96). These empirically estimated exponents provided an improved description of the observed data relative to the corresponding fixed exponents of 0.75 for CL/F and 1.0 for Vc/F within the current dataset, based on objective function comparisons (*p* < 0.01) [13]. Lean body mass and body mass index were also evaluated as alternative body-size descriptors, but neither improved model performance nor yielded greater model stability than total body weight. The effects of body weight on the apparent peripheral volume of distribution (Vp/F) and apparent intercompartmental clearance (Q/F) were also formally evaluated using the same allometric framework. However, inclusion of body weight on either Vp/F or Q/F did not produce a statistically meaningful improvement in model fit (ΔOFV < 3.84 for both), and parameter estimates for these relationships were imprecise (RSE > 50%). This likely reflects the limited information available to characterize peripheral disposition parameters in a dataset that contained a substantial proportion of sparse pharmacokinetic sampling from the Phase II and Phase III studies.

In addition, comparison between the model applying fixed standard exponents to all four parameters and that applying freely estimated exponents to all four parameters showed that extending allometric scaling to Q/F and Vp/F did not materially improve prediction- and variability-corrected VPC performance (Supplementary Figures S2 and S3). These findings support the adequacy of the final model within the studied population, but do not establish the universal superiority of any single allometric specification. Accordingly, body weight effects were retained only on CL/F and Vc/F in the final model to preserve parsimony and numerical stability.

Exploratory assessment of individual *post hoc* estimates of relative bioavailability, together with prior noncompartmental analysis, indicated that pixavir exposure increased less than dose proportionally over the 10–160 mg dose range. Dose was therefore evaluated as a covariate for relative bioavailability (F) using linear, power, and hyperbolic functions. A Michaelis–Menten-type model was additionally explored as a mechanistic alternative. A hyperbolic function best described the dose effect on relative bioavailability:

$$F=\frac{\theta }{\theta +DOSE/40}$$

where DOSE is the administered dose normalized to the 40 mg reference dose. This function was selected based on the largest improvement in model fit (ΔOFV = −115.2), acceptable parameter precision (RSE = 13.5%), and its ability to capture the observed decline in apparent bioavailability with increasing dose. This pattern is consistent with a dose-dependent absorptive and/or presystemic process, although the model should be interpreted as empirical rather than mechanistic.

Compared with the empirical bioavailability model, the Michaelis–Menten formulation resulted in a substantially higher OFV and showed poor numerical stability, indicating inadequate parameter identifiability in the current dataset. These findings suggest that the model was overparameterized relative to the information content of the available data, and the corresponding parameters could not be estimated reliably. Therefore, the Michaelis–Menten model was not retained.

A high-fat meal reduced *k*_a_; however, its effect on overall systemic exposure, including AUC_inf_ and C_max_, was limited and was not considered clinically meaningful in the target patient population. No statistically significant effects of age, sex, or influenza virus type on pixavir pharmacokinetics were identified. Shrinkage estimates for all random effects were below 20%, supporting the robustness of the model and the reliability of the empirical Bayes estimates.

**Figure 1:**
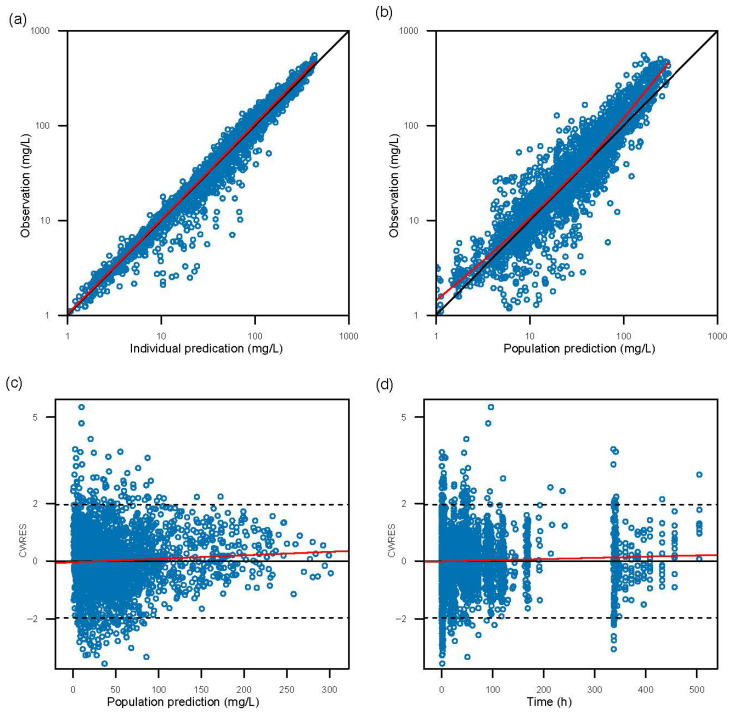
Goodness-of-fit of the final population pharmacokinetic model for pixavir. (**a**) Observed concentrations vs. individual predicted concentrations; (**b**) Observed concentrations vs. population predicted concentrations; (**c**) Conditional weighted residuals (CWRES) vs. population predicted concentrations; (**d**) Conditional weighted residuals (CWRES) vs. time after the first dose. In panels (**a**,**b**), the red lines represent locally estimated scatterplot smoothing (LOESS) lines, and the reference lines denote the line of identity (x = y). In panels (**c**,**d**), the red lines represent linear regression lines, and the reference lines denote y = 0.

**Figure 2:**
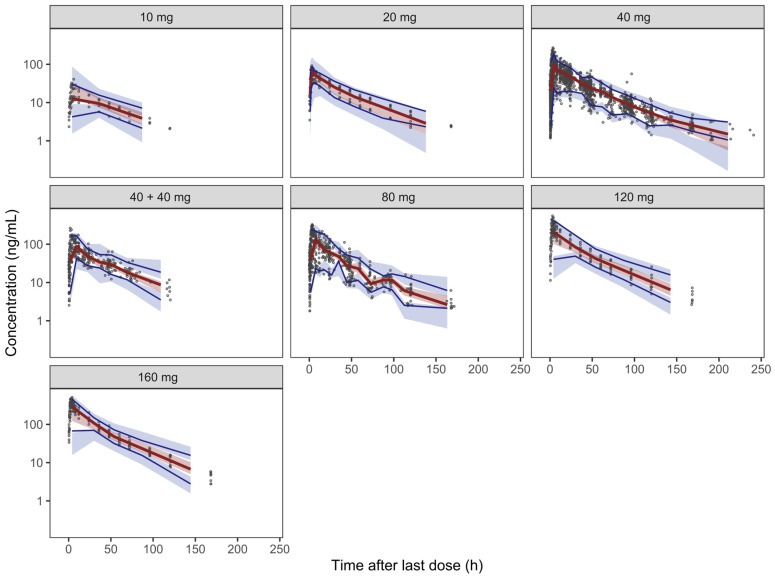
Visual predictive check (VPC) of the final population pharmacokinetic model for pixavir, stratified by dose group: 10, 20, 40, 80, 120, and 160 mg (single-dose cohorts) and 40 mg administered twice (40 + 40 mg). Black circles represent observed concentrations. The solid red line denotes the observed median (50th percentile), and the shaded band between the lower and upper observed percentile lines represents the observed 5th–95th percentile interval. The blue simulation-based bands indicate the 95% confidence intervals (CIs) of the corresponding simulated percentiles (5th, 50th, and 95th) generated from 1000 Monte Carlo simulations. Agreement between observed percentile curves and simulated percentile CIs indicates adequate predictive performance of the model across dose strata.

Goodness-of-fit plots (Figure 1) demonstrated good agreement between observed concentrations and both population- and individual-predicted values. Conditional weighted residuals were symmetrically distributed across time and predicted concentration ranges, with no indication of time-dependent bias. Visual predictive checks (Figure 2) showed that observed percentiles of pixavir concentrations fell within the simulated prediction intervals. Additionally, nonparametric bootstrap analysis yielded stable parameter estimates, further supporting the robustness of the final model. Parameter estimates for this model are presented in Table 3.

As a further check on model plausibility, PK parameters derived by noncompartmental analysis (NCA) from the rich-sampling Phase I cohort were compared with the corresponding estimates from the final population PK model (Supplementary Table S1). The NCA-derived CL/F and terminal half-life were broadly consistent with the model-based estimates, supporting the internal consistency of the two approaches. In addition, the extent of distribution estimated by NCA was similar in magnitude to that implied by the final model parameters, as reflected by the combined apparent central and peripheral volumes of distribution. Collectively, these findings provide additional checks on the final model and support its structural adequacy.

The final PopPK model is described below:

$$\mathrm{CL}(L/h)=9.14\times {\left(\frac{WT}{61.30}\right)}^{0.961}$$

$$\;\;\;\;V_{c}(L)=268\times {\left(\frac{WT}{61.30}\right)}^{1.69}$$

Q(L/h)=5.28

Vp(L)=101

$$k_{a}\left(\frac{1}{h}\right)=\left\{\begin{array}{c} 0.56,\;\;\;fasting\\ 0.34,\;\;\;standard\;diet\\ 0.116,\;\;\;\mathrm{high}\;\mathrm{fat}\;meal \end{array}\right.$$

$$F\;(\mathrm{Relative}\;\mathrm{bioavailability})=\frac{3.13}{3.13+DOSE/40}$$

#### 3.1.2. Model Applications

Simulations using the final PopPK model were conducted to evaluate pixavir exposure over a body weight range of approximately 40–120 kg under three dosing scenarios: fixed 40 mg, fixed 80 mg, and the approved weight-based regimen (40 mg for <80 kg; 80 mg for ≥80 kg). As shown in Supplementary Figure S4, a fixed 40 mg dose resulted in lower predicted AUCinf and C_24h_ in patients weighing ≥80 kg, whereas the weight-based regimen yielded broadly comparable exposure across body weight groups, supporting the 80 kg dosing cutoff.

The impact of food status on key exposure metrics was also examined (Supplementary Figure S5). The food effect was not judged clinically meaningful within the context of the observed exposure–response data.

### 3.2. Exposure–Response Analysis

A total of 408 individuals, including 359 adults and 49 adolescents, were included in the ER analysis, all of whom had both exposure metrics and evaluable clinical or virological endpoints. Adolescents accounted for approximately 12% of the population and had a lower mean body weight than adults (58.42 kg vs. 64.71 kg). The demographics of the enrolled subjects and their pharmacokinetic (PK) exposure levels are summarized in Table 4 and Table 5, respectively.

#### 3.2.1. Exposure–Efficacy Analysis

For the primary clinical endpoint, time to alleviation of influenza-related symptoms, no meaningful exposure–efficacy relationship was identified. An Emax model was initially evaluated but did not provide stable or identifiable parameter estimates. Log-linear models also failed to improve model fit relative to a simple linear model, based on AIC, BIC, and goodness-of-fit diagnostics. The linear model was therefore retained as the most parsimonious description, with slope estimates close to zero and 95% confidence intervals including zero.

**Figure 3:**
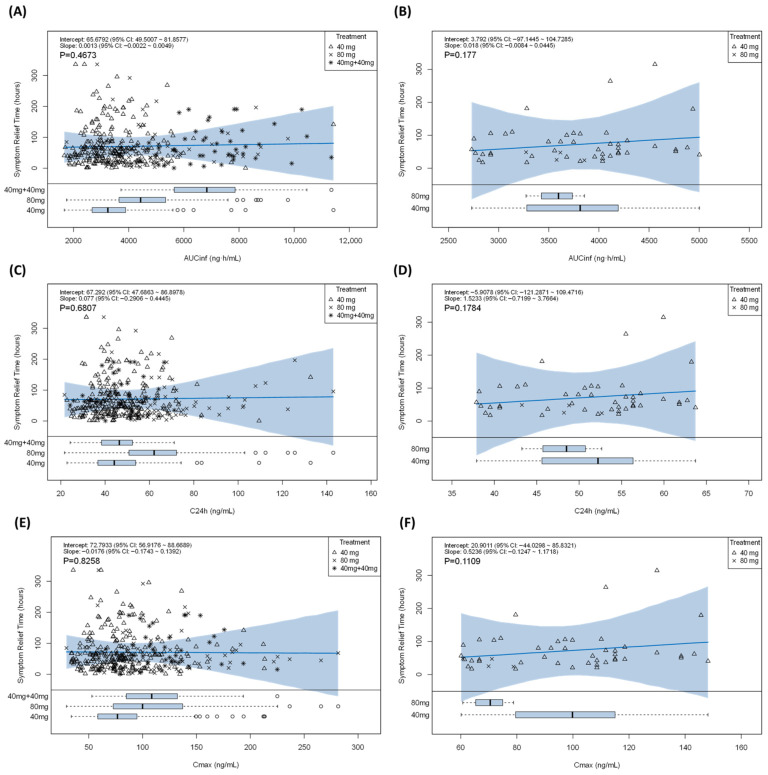
Scatterplots depicting the relationship between the time to alleviation of influenza-related symptoms (TTAS) and exposure metrics in adults and adolescents, stratified by dose group (40 mg, 80 mg single-dose cohorts, and 40 mg administered twice [40 mg + 40 mg]). (**A**) Adults: TTAS vs. AUC_inf_; (**B**) Adolescents: TTAS vs. AUC_inf_; (**C**) Adults: TTAS vs. C_24h_; (**D**) Adolescents: TTAS vs. C_24h_; (**E**) Adults: TTAS vs. C_max_; (**F**) Adolescents: TTAS vs. C_max_. The solid line is the fitted regression line, and the shaded area represents the 95% confidence interval (CI) of the fitted mean. The slope estimate and its 95% CI are shown in the in-panel annotations.

Consistent with the model results, visual inspection showed substantial overlap in symptom alleviation times across the full range of exposures in both adults and adolescents (Figure 3). Higher exposure was not associated with faster symptom alleviation. Sensitivity analyses stratified by age group, body weight tertiles, and baseline symptom score tertiles yielded consistent findings, with no discernible exposure–efficacy trend in any subgroup (Supplementary Figures S6–S8). Overall, these results indicate that, within the exposure range studied, pixavir efficacy was not exposure-dependent.

Similarly, no consistent trends were observed between exposure metrics and virological responses. Reductions in viral titers at day 2 were comparable across all exposure levels in both adults and adolescents under the proposed dosing regimen.

#### 3.2.2. Exposure–Safety Analysis

Safety evaluations were conducted in the entire pooled trial population (500 pixavir-treated and 250 placebo-treated individuals), whereas the ER analysis was restricted to the subset with available PK and endpoint data (*n* = 408). Pixavir marboxil demonstrated a generally favorable safety profile in the study. Adverse events were reported in 25.0% of participants receiving pixavir marboxil (125 out of 500 individuals) compared to 28.8% of those in the placebo group (72 out of 250 individuals), with most events being mild to moderate in severity. The most frequently reported adverse events in the pixavir marboxil group, with an incidence greater than 1.0%, included decreased neutrophil count, hyperuricemia, sinus arrhythmia, and vomiting.

Exploratory analyses showed no apparent difference in safety event rates between pixavir marboxil and placebo and no trend across exposure strata. Therefore, the prespecified criteria for proceeding to formal regression-based safety ER modeling were not met, and no further safety ER modeling was performed.

## 4. Discussion

This study provides the first comprehensive analysis of the PopPK and ER relationships of pixavir marboxil, a novel anti-influenza virus agent, in both adult and adolescent patients with influenza. The analysis defines the principal determinants of pixavir exposure, supports the current body weight-based dosing strategy, and indicates that neither efficacy nor safety varied meaningfully across the exposure range achieved with the studied regimens.

Body weight was the most important intrinsic covariate influencing pixavir pharmacokinetics. Incorporation of body weight as an allometric covariate significantly improved model fit and reduced inter-individual variability in both CL/F and Vc/F, indicating that body size is a clinically relevant determinant of systemic exposure. Heavier individuals had higher CL/F and Vc/F, which is directionally consistent with established pharmacokinetic principles linking drug disposition to body size [15]. The empirically estimated exponent for CL/F (0.961) was higher than the conventional theoretical value of 0.75, whereas that for Vc/F (1.69) exceeded the canonical value of 1.0.

Although canonical allometric exponents are widely used as physiologically motivated reference values, their applicability in within-species human PopPK analyses remains context-dependent [10]. In the present study, the estimated exponents should therefore be interpreted as empirical approximations that adequately described the observed data within the studied population, rather than as universally generalizable physiological constants. Nevertheless, they suggest that body size influences CL/F and Vc/F in pixavir at least as strongly as predicted by standard allometric assumptions.

Mechanistically, the near-linear scaling of CL/F with body weight may be compatible with elimination pathways involving glucuronidation and fecal excretion, whereas the steeper-than-expected increase in Vc/F may reflect distribution characteristics related to the compound’s high plasma protein binding and its tissue partitioning. Regardless of mechanism, these findings provide quantitative support for the currently recommended weight-based regimen (40 mg for <80 kg; 80 mg for ≥80 kg), which appears to compensate for body size-related increases in CL/F and Vc/F and thereby helps maintain broadly comparable exposure across the studied weight range.

Food, particularly a high-fat meal, reduced *k*_a_, indicating an effect primarily on the rate rather than the extent of absorption. Accordingly, fed administration was associated with delayed absorption and a lower C_max_, whereas the impact on overall systemic exposure was modest, with an approximately 19% reduction in AUC_inf_ in the present dataset. The larger decrease in C_max_ (41%) is therefore most consistent with slower absorption rather than a clinically meaningful reduction in bioavailability. The clinical relevance of this food effect appears limited. No exposure–response relationship was identified for AUC_inf_, C_max_, or C_24h_ for any efficacy endpoint across the observed exposure range. These data indicate that moderate food-related changes in total exposure or peak concentration are unlikely to alter antiviral response. This interpretation is also mechanistically plausible, as antiviral activity of PA endonuclease inhibitors may depend more on sustained effective exposure than on C_max_, particularly given the prolonged target residence time of pixavir [16,17]. Under this framework, a reduction in C_max_ would not be expected to meaningfully compromise efficacy. Taken together, the PK and exposure–response findings indicate that the observed food effect is unlikely to be clinically meaningful and support administration of pixavir marboxil without regard to food intake.

A further notable finding was the less-than-dose-proportional increase in exposure over the 10–160 mg dose range, which was best described by a dose effect on apparent bioavailability. The available clinical data do not allow a definitive mechanistic attribution. Plausible explanations include saturable presystemic metabolism, saturable intestinal transport, and/or formulation-related dissolution or solubility limitations at higher doses. Distinguishing among these possibilities would require dedicated mechanistic in vitro and clinical studies. Importantly, this limitation does not undermine the exposure–response analyses, because all individual exposure metrics used in those analyses were derived from the final population PK model and therefore already incorporated the dose-dependent bioavailability term for each patient.

The absence of a detectable exposure–efficacy gradient within the studied exposure range should not be interpreted as evidence that exposure is irrelevant to dose selection. Rather, the flat E-R relationship suggests that the evaluated regimens achieved exposures on or near the plateau of the concentration-effect curve, such that variation within this range did not translate into detectable differences in outcome. This is consistent with the lack of a stable E_max_ model and the near-zero slopes in the linear analyses, but does not imply that lower exposures would be equally effective, because the lower boundary of the efficacious range was not defined.

Accordingly, the rationale for weight-based dosing is not to exploit an exposure–efficacy gradient, but to reduce the risk of underexposure in heavier patients with systematically higher CL/F and Vc/F. PopPK simulations showed that, with a fixed 40 mg dose, patients weighing ≥80 kg had AUC_inf_ values approximately 25–35% lower than those weighing <80 kg. The weight-based regimen (40 mg for <80 kg; 80 mg for ≥80 kg) therefore helps maintain comparable exposures across the studied weight range.

This view is supported, albeit indirectly, by translational comparison with in vitro antiviral potency. Pixavir demonstrated potent in vitro antiviral activity, with low-nanomolar IC_50_ values in polymerase acidic (PA) endonuclease inhibition assays and low-nanomolar EC_50_ values in MDCK cell-based plaque reduction assays against both influenza A and B [1]. As an exploratory exercise, the reported EC_50_ values were converted to total plasma concentration equivalents after correction for the high plasma protein binding of pixavir (>96%), yielding approximate thresholds of 4.73–41.76 ng/mL for influenza A and 25.68–37.16 ng/mL for influenza B based on the MDCK assay [1]. The geometric mean C_max_ and C_24h_ values achieved with the 40/80 mg regimen in adults and adolescents were generally above these values, and AUC_inf_ likewise suggested sustained exposure within or above the putative antiviral activity range. Although such comparisons are inherently approximate and should not be interpreted as defining a formal human target exposure or PK/PD cutoff, they provide supportive context that the studied regimen achieves pharmacologically relevant exposure [18,19]. Taken together, these findings support dose adequacy and do not suggest a need for further dose escalation.

From a safety perspective, no meaningful relationship was observed between pixavir exposure and the incidence of adverse events. The overall tolerability profile was favorable, with most adverse events being mild to moderate. In particular, decreased neutrophil count and hyperuricemia were generally mild and were not associated with increasing exposure. Although the mechanisms underlying these findings remain to be clarified, the absence of exposure dependence argues against a narrow exposure-driven toxicity window within the studied range. These results support the clinical utility of pixavir marboxil and suggest that the recommended regimen is well tolerated, with no evidence of excess safety risk at higher exposure levels.

Comparison with other anti-influenza agents should be made cautiously; nevertheless, several findings are noteworthy. Like baloxavir marboxil, pixavir marboxil is an orally administered cap-dependent endonuclease inhibitor developed for single-dose treatment and is subject to food effects. Food intake reduces the C_max_ and AUC_inf_ of baloxavir marboxil by approximately 48% and 36%, respectively [20], whereas the corresponding reductions with pixavir are somewhat smaller, at 41% and 19%. In addition, pixavir has a markedly shorter median terminal half-life than baloxavir acid (36 h vs. 79.1–93.9 h) [20]. However, whether these pharmacokinetic differences translate into clinically meaningful differences in onset or duration of antiviral activity, resistance selection, or tolerability cannot be determined from the present analysis. Similarly, cross-study comparisons of safety should be interpreted cautiously given differences in study design, patient populations, and adverse event ascertainment.

This study has several limitations. First, all participants were enrolled in China and were Chinese, which may limit extrapolation to other racial or ethnic populations. Second, the studied population had a relatively narrow range of age, body weight, and BMI; therefore, the identified covariate relationships should be interpreted within the observed data range and should not be extrapolated directly to older adults, patients with obesity, children younger than 12 years of age, or other clinically important subgroups without prospective evaluation. Third, the adolescent subgroup was relatively small, with fewer younger adolescents and high-body-weight individuals included, which may slightly limit the representativeness of this group. Fourth, although the exposure–response analyses supported dose adequacy within the evaluated regimens, they did not establish a formal PK/PD target or fully characterize the concentration–effect relationship. Finally, the virological sampling in the available dataset was relatively limited and inconsistent, which may affect the ability to fully describe viral dynamics; future studies with more comprehensive virological sampling could provide additional insight into the PK-virological relationship.

## 5. Conclusions

This analysis provides a quantitative basis for describing pixavir PK and supporting dose characterization in Chinese adults and adolescents with acute influenza. Body weight was the primary determinant of exposure and supports the current weight-based dosing strategy. Although food reduced the rate of absorption and exposure increased less than dose-proportionally, neither finding appeared clinically meaningful in the context of the observed exposure–response data. The absence of exposure-dependent efficacy or safety signals within the studied range supports the adequacy of the approved regimen in the studied population. Extrapolation beyond this population and mechanistic interpretation of the nonlinear bioavailability process, however, should be approached with caution.

## Figures and Tables

**Table 1:** Summary of clinical studies included in the population pharmacokinetic analysis.

Study ID	Study Design	Dosing Regimen	Sampling Strategy	No. of Subjects/PK Samples
TG-1000-C-01	Phase I, single-center, randomized, double-blind, placebo-controlled, single ascending dose study evaluating safety, tolerability, and pharmacokinetics of TG-1000 in healthy volunteers; included food effect evaluation	Part A: Single ascending doses of 10, 20, 40, 80, 120, and 160 mg (*n* = 44) Part B: Two-way crossover food effect study, 40 mg (*n* = 12)	Pre-dose and at 0.5, 1, 1.5, 2, 2.5, 3, 3.5, 4, 5, 6, 8, 12, 24, 36, 48, 60, 72, 96, 120, and 168 h post-dose. additional 336 h post-dose sampling for 40, 80, 120, and 160 mg groups	Healthy adults (56/1331)
TG-1000-C-02	Phase II, multicenter, randomized, double-blind, dose-ranging study comparing TG-1000 to placebo in uncomplicated acute influenza infection	Single dose 40 mg (*n* = 51) or 80 mg (*n* = 48), or two doses of 40 mg each administered 48 h apart (*n* = 48)	Sparse sampling: 0.5–4 h, Day 2, Day 3, Day 5 post-dose Intensive sampling: 0, 1, 2, 4, 8, 12, 24, 48 h post-dose for the first dose and 1, 2, 4, 8, 12, 24, 48, 72, 120 h post-dose for the second dose	Adults with uncomplicated acute influenza infection (147/868)
TG-1000-C-03	Phase III, multicenter, randomized, double-blind study evaluating efficacy and safety of TG-1000 vs. placebo in adults and adolescents with uncomplicated acute influenza	Single oral dose of 40 mg for patients weighing 40–80 kg (*n* = 192) or 80 mg for patients ≥ 80 kg (*n* = 28)	Sparse sampling: 0.5–4 h, Day 2, Day 3, Day 5 post-dose Intensive sampling: Pre-dose, 1, 2, 4, 8, 12, 24, 48, 72, 96, 120, 144, 168, 192, 216, 240 h post-dose	Adults and adolescents (≥12 years) with uncomplicated acute influenza (220/926)

**Table 2:** Summary of demographic and baseline characteristics.

Characteristic	Category	TG-1000-C-01 (*n* = 56)	TG-1000-C-02 (*n* = 147)	TG-1000-C-03 (*n* = 220)	Total (*n* = 423)
Disease	Healthy	56	0	0	56 (13.2%)
	Patients with influenza	0	147	220	367 (86.8%)
Sex	Female	28	61	101	190 (44.9%)
	Male	28	86	119	233 (55.1%)
Treatment regimen	10 mg	4	0	0	4 (0.9%)
	20 mg	8	0	0	8 (1.9%)
	40 mg	20	51	192	263 (62.2%)
	80 mg	8	48	28	84 (19.9%)
	120 mg	8	0	0	8 (1.9%)
	160 mg	8	0	0	8 (1.9%)
	40 mg + 40 mg	0	48	0	48 (11.3%)
Food intake	Fasted state	56	68	9	133 (31.4%)
	Standard diet	0	79	211	290 (68.6%)
	High-fat meal	12	0	0	12 (2.8%)
Age (years)		23.3 (5.08)	29.4 (8.86)	26.6 (7.72)	27.1 (8.08)
Weight (kg)		58.4 (5.56)	64.1 (12.0)	65.0 (12.6)	63.8 (11.9)
Height (cm)		165 (6.90)	168 (9.06)	169 (8.55)	168 (8.64)
Body mass index (kg/m^2^)		21.5 (1.25)	22.6 (3.06)	22.6 (3.19)	22.4 (2.97)
Total protein (g/L)		74.2 (3.59)	75.8 (4.06)	76.6 (6.23)	76.0 (5.31)
Alanine aminotransferase (U/L)		16.0 (8.91)	22.9 (18.7)	21.8 (19.4)	21.4 (18.2)
Total bilirubin (μmol/L)		10.9 (4.46)	10.2 (5.25)	11.2 (5.50)	10.8 (5.30)
Serum creatinine (μmol/L)		126 (24.9)	122 (31.6)	138 (38.4)	131 (35.3)

Continuous variables were expressed as mean (standard deviation), and categorical variables were expressed as the number or number with percentage.

**Table 3:** Parameter estimates of the final population pharmacokinetic model and bootstrap evaluation.

Parameter	Description	Estimate	Relative Standard Error (%)	Bootstrap Median (2.5%, 97.5%)
Fixed Effects				
CL/F (L/h)	Apparent clearance	9.14	4.0	9.12 (8.18, 10.12)
CL_WT	Allometric exponent for body weight on CL/F	0.961	8.2	0.961 (0.805, 1.13)
Vc/F (L)	Central volume of distribution	268	4.9	267 (231, 304)
Vc_WT	Allometric exponent for body weight on Vc/F	1.69	8.5	1.69 (1.43, 1.96)
Vp/F (L)	Peripheral volume of distribution	101	6.4	101 (85.8, 117)
Q/F (L/h)	Inter-compartmental clearance	5.28	11.9	5.26 (4.04, 6.86)
*k*_a_ (1/h)	Absorption rate for fasting	0.56	10.7	0.559 (0.497, 0.63)
*k*_a_ (1/h)	Absorption rate for standard diet	0.34	6.1	0.34 (0.297, 0.390)
*k*_a_ (1/h)	Absorption rate for high fat meal	0.116	10.1	0.116 (0.08, 0.154)
ALAG (h)	Lag time	0.333	1.4	0.333 (0.313, 0.35)
F_dose_	Effect of dose on relative bioavailability	3.13	13.5	3.11 (2.19, 4.73)
Inter-individual variability (IIV, %)				
*η k*_a_	IIV of *k*_a_	75.8	4.7	75.59 (69.21, 83.11)
*η*_CL/F_	IIV of CL/F	26.6	3.9	26.57 (24.08, 29.1)
*η*_Vc_	IIV of Vc/F	39.1	5.4	39.08 (34.0, 43.8)
CL/F-Vc/F	Covariance between IIV of CL/F and V_C_/F	0.0928	9.4	0.0925 (0.0725, 0.115)
Residual unexplained variability				
ε	Proportional error (%)	21.95	0.7	21.86 (19.97, 23.56)

IIV, inter-individual variability; CL/F, apparent clearance; Vc/F, apparent central volume of distribution; Vp/F, apparent peripheral volume of distribution; Q/F, apparent intercompartmental clearance; *k*_a_, absorption rate constant; ALAG, absorption lag time.

**Table 4:** Demographics of subjects for exposure–response analysis.

Characteristics	Statistics/Category	Adults (*n* = 359)	Adolescents (*n* = 49)
Study	TG-1000-C-02 [count (%)]	147 (40.9%)	0 (0%)
	TG-1000-C-03 [count (%)]	212 (59.1%)	49 (100%)
Sex	Female/Male	159/200	23/26
Age (years)	Mean (standard deviation)	27.03 (8.41)	15.20 (1.57)
Weight (kg)	Mean (standard deviation)	64.71 (12.38)	58.42 (14.65)
Body mass index (kg/m^2^)	Mean (standard deviation)	22.58 (3.14)	20.74 (3.54)
Influenza symptom score	Mean (standard deviation)	10.89 (2.98)	11.43 (2.47)
Virus Type	A [count (%)]	200 (55.7%)	35 (71.4%)
	B [count (%)]	159 (44.3%)	13 (26.5%)
	A and B (co-infection) [count (%)]	0 (0%)	1 (2%)
Dosing Regimen	40 mg [count (%)]	234 (65.2%)	45 (91.8%)
	80 mg [count (%)]	77 (21.4%)	4 (8.2%)
	40 mg + 40 mg [count (%)]	48 (13.4%)	0 (0%)

**Table 5:** Summary of the exposure metrics for patients included in the exposure–response analysis.

Population and Dose Group	Statistics	AUC_inf_ (ng·h/mL)	C_24h_ (ng/mL)	C_max_ (ng/mL)
Adults (*n* = 359)				
40 mg (*n* = 234)	Geometric Mean (gCV%)	3236.28 (30.3)	44.45 (28.09)	75.81 (40.05)
	Median (min-max)	3245.87 (1666.84–11,423.16)	44.1 (22.79–132.7)	76.6 (33.86–213.3)
80 mg (*n* = 77)	Geometric Mean (gCV%)	4522.93 (33.33)	61.94 (33.91)	101.26 (47.97)
	Median (min-max)	4432.52 (1749.37–9773.13)	62.11 (21.68–142.94)	100 (29.33–281.53)
40 mg + 40 mg (*n* = 48)	Geometric Mean (gCV%)	6716.98 (25.49)	45.05 (24.47)	107.77 (32.62)
	Median (min-max)	6834.34 (3722.34–11,349.81)	46.41 (24.24–71.22)	108.5 (52.93–225.04)
Adolescents (*n* = 49)				
40 mg (*n* = 45)	Geometric Mean (gCV%)	3721.09 (18.01)	50.54 (15.77)	95.95 (27.27)
	Median (min-max)	3813.18 (2731.06–5002.44)	52.22 (37.89–63.72)	99.83 (60.14–148.16)
80 mg (*n* = 4)	Geometric Mean (gCV%)	3574.8 (6.74)	48.12 (8.13)	69.84 (10.75)
	Median (min-max)	3597.07 (3273.7–3855.52)	48.51 (43.25–52.67)	70.52 (60.72–78.8)

gCV%: geometric Coefficient of Variation%.

## Data Availability

The data presented in this study are available on request from the corresponding author due to confidentiality agreements with the sponsoring company.

## Supplementary Materials

The following supporting information can be downloaded at: https://www.mdpi.com/article/10.3390/pharmaceutics18050550/s1, Table S1. Comparison of noncompartmental analysis (NCA)-derived pharmacokinetic parameters from the rich-sampling Phase I cohorts with the corresponding typical estimates from the final population pharmacokinetic model. Figure S1. Individual and median observed pixavir concentration–time profiles across dose groups on linear and semi-logarithmic scales. Figure S2. Prediction- and variability-corrected Visual Predictive Check for the model with standard exponents to all four parameters stratified by weight. Figure S3. Prediction- and variability-corrected Visual Predictive Check for the model with freely estimated exponents to all four parameters stratified by weight. Figure S4. Model-based simulations of pixavir exposure across body weight under three dosing scenarios: fixed 40 mg, fixed 80 mg, and the weight-based regimen (40 mg for <80 kg and 80 mg for ≥80 kg). Figure S5. Forest plot showing the effect of food status on key pixavir exposure metrics relative to the overall exposure distribution. Figure S6. Sensitivity analysis of the exposure–efficacy relationship for time to alleviation of influenza-related symptoms, stratified by age group (adults versus adolescents). Figure S7. Sensitivity analysis of the exposure–efficacy relationship for time to alleviation of influenza-related symptoms, stratified by body weight. Figure S8. Sensitivity analysis of the exposure–efficacy relationship for time to alleviation of influenza-related symptoms, stratified by baseline influenza symptom score. The label indicates pixavir marboxil.

## Abbreviations

The following abbreviations are used in this manuscript:

AUC_inf_	Area under the concentration–time curve extrapolated to infinity
CL/F	Apparent clearance
C_max_	Maximum plasma concentration
C_24h_	Plasma concentration at 24 h post-dose
CWRES	Conditional weighted residuals
ER	Exposure–response
FOCE-I	First-order conditional estimation with interaction
GOF	Goodness-of-fit
IIV	Inter-individual variability
*k*_a_	First-order absorption rate constant
LLOQ	Lower limit of quantification
PK	Pharmacokinetics
PopPK	Population pharmacokinetics
RSE	Relative standard error
RUV	Residual unexplained variability
SD	Standard deviation
TCID_50_	50% tissue culture infectious dose
TRAE	Treatment-related adverse event
Vc/F	Apparent central volume of distribution
VPC	Visual predictive check
