# Supplementary material for: Population Pharmacokinetics and Exposure–Response Analysis of Oral Pixavir Marboxil in Adults and Adolescents with Influenza
Source: Pharmaceutics. 2026 Apr 30;18(5):550. doi: 10.3390/pharmaceutics18050550 (PMC13210205; doi:10.3390/pharmaceutics18050550)
Supplement: Supplementary file 1 [file pharmaceutics-18-00550-s001.zip › Figure S4-WT.pdf]

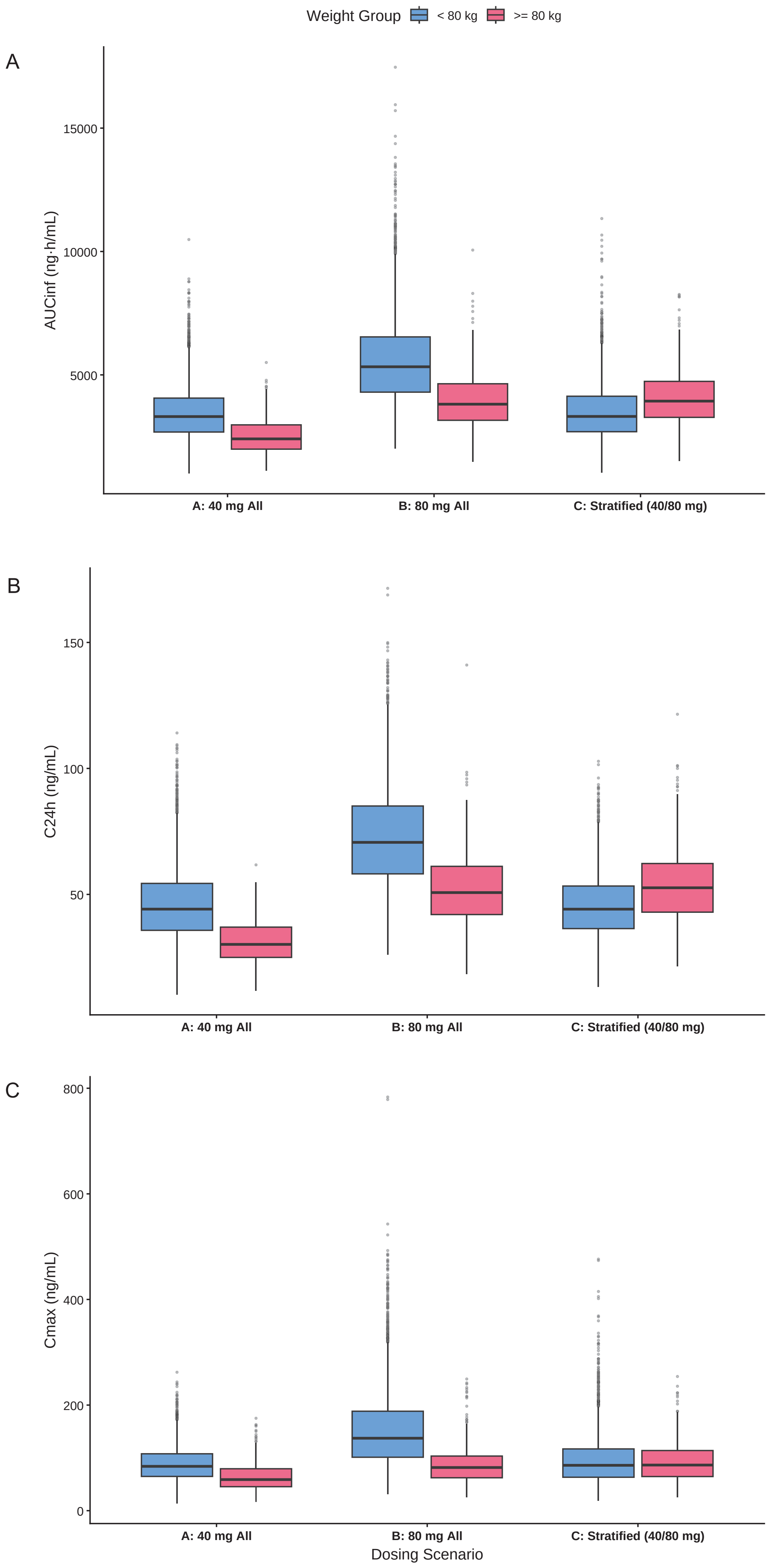

Figure S4. Model-based simulations of pixavir exposure across body weight under three dosing scenarios: fixed 40 mg, fixed 80 mg, and the weight-based regimen (40 mg for <80 kg and 80 mg for ≥80 kg). A: AUCinf; B: C24h; C: Cmax
