# Supplementary material for: Population Pharmacokinetics and Exposure–Response Analysis of Oral Pixavir Marboxil in Adults and Adolescents with Influenza
Source: Pharmaceutics. 2026 Apr 30;18(5):550. doi: 10.3390/pharmaceutics18050550 (PMC13210205; doi:10.3390/pharmaceutics18050550)
Supplement: Supplementary file 1 [file pharmaceutics-18-00550-s001.zip › Figure S5-food.pdf]

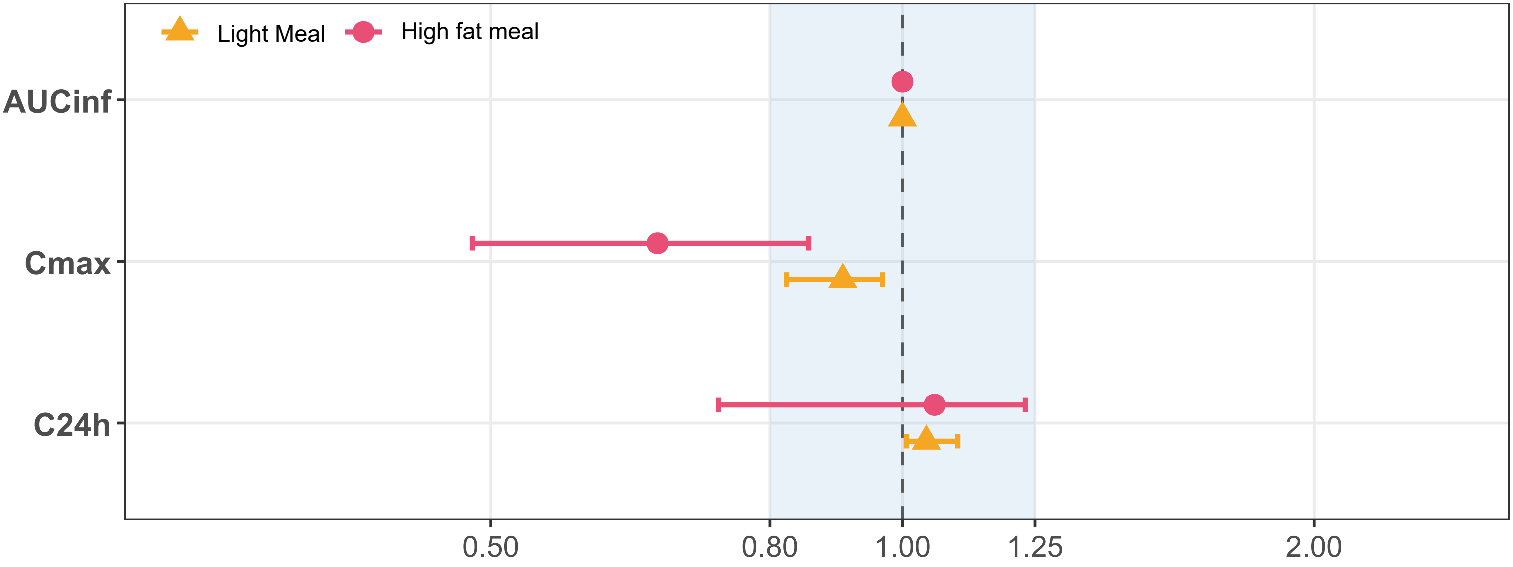

Figure S5. Forest plot showing the effect of food status on key pixavir exposure metrics relative to the overall exposure distribution.
Geometric mean ratio vs fasted (point) with 5th–95th percentile interval (bar)[shaded region: 80–125% bioequivalence zone]
