# Supplementary material for: Population Pharmacokinetics and Exposure–Response Analysis of Oral Pixavir Marboxil in Adults and Adolescents with Influenza
Source: Pharmaceutics. 2026 Apr 30;18(5):550. doi: 10.3390/pharmaceutics18050550 (PMC13210205; doi:10.3390/pharmaceutics18050550)
Supplement: Supplementary file 1 [file pharmaceutics-18-00550-s001.zip › Figure S6-age.pdf]

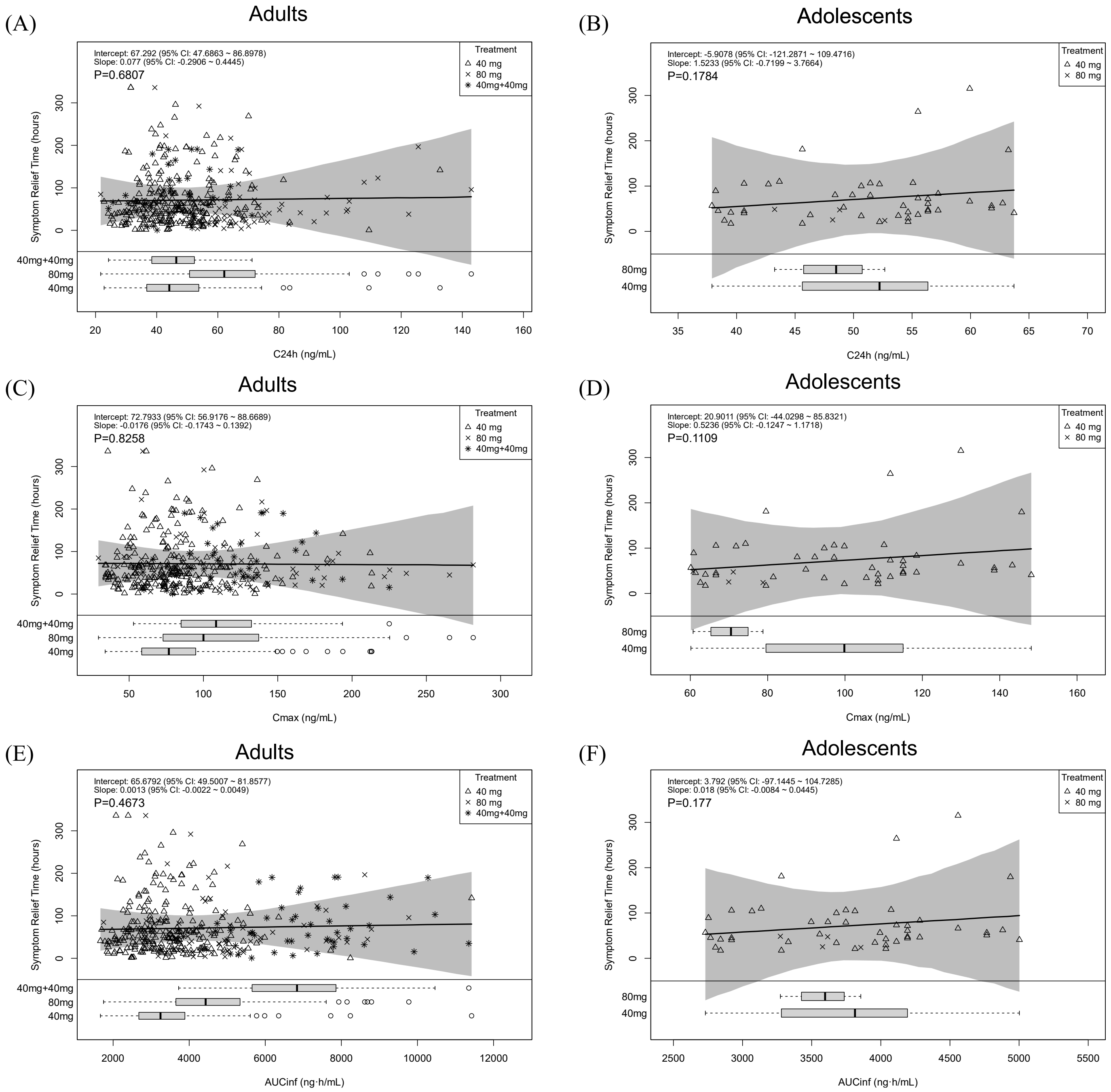

Figure S6. Sensitivity analysis of the exposure–efficacy relationship for time to alleviation of influenza-related symptoms, stratified by age group (adults versus adolescents).

(A) adults, exposure–efficacy relationship based on C24h;
(B) adolescents, exposure–efficacy relationship based on C24h;
(C) adults, exposure–efficacy relationship based on Cmax;
(D) adolescents, exposure–efficacy relationship based on Cmax;
(E) adults, exposure–efficacy relationship based on AUCinf;
(F) adolescents, exposure–efficacy relationship based on AUCinf.
