# Supplementary material for: Population Pharmacokinetics and Exposure–Response Analysis of Oral Pixavir Marboxil in Adults and Adolescents with Influenza
Source: Pharmaceutics. 2026 Apr 30;18(5):550. doi: 10.3390/pharmaceutics18050550 (PMC13210205; doi:10.3390/pharmaceutics18050550)
Supplement: Supplementary file 1 [file pharmaceutics-18-00550-s001.zip › Figure S7-wt.pdf]

(A) adults: ≤ 80kg

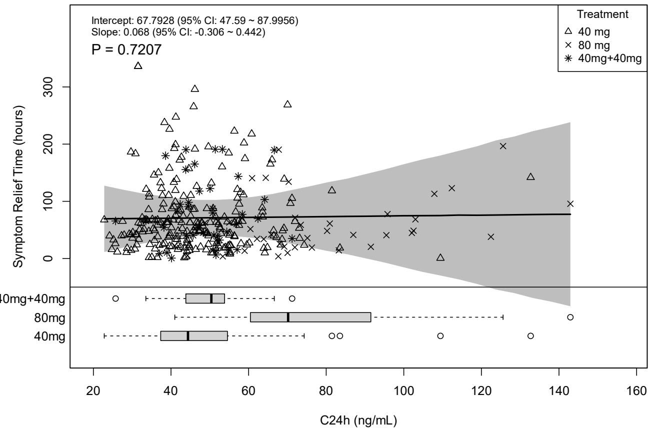

(B) adults: > 80kg

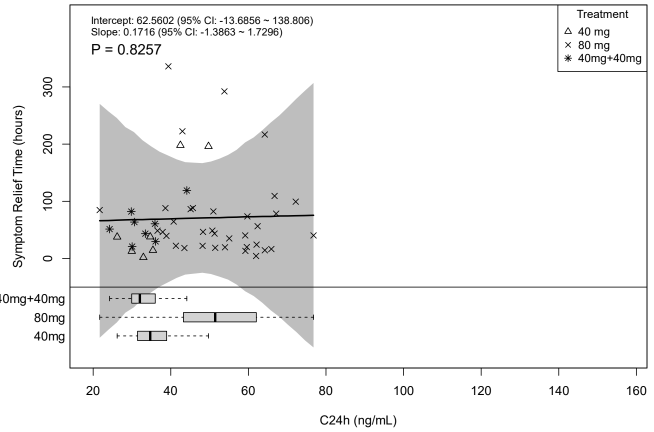

(C) adults: ≤ 80kg

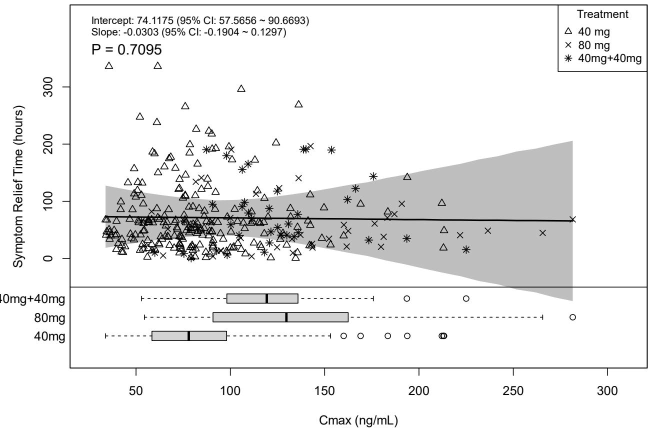

(D) adults: > 80kg

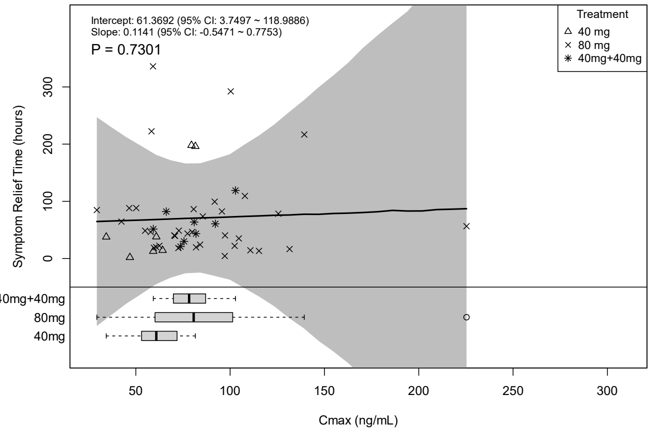

(E) adults: ≤ 80kg

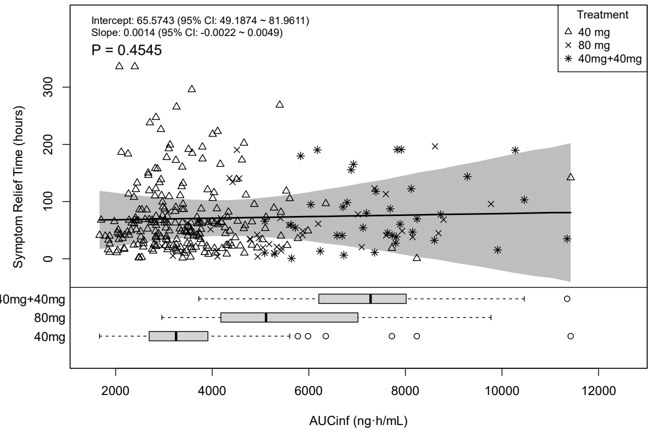

(F) adults: > 80kg

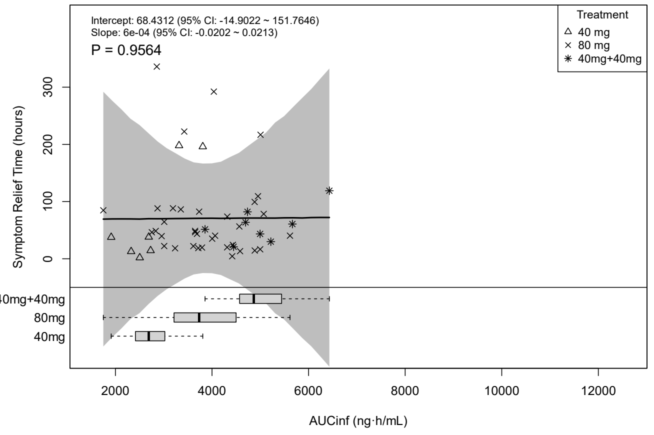

(G) adolescents: ≤ 80kg

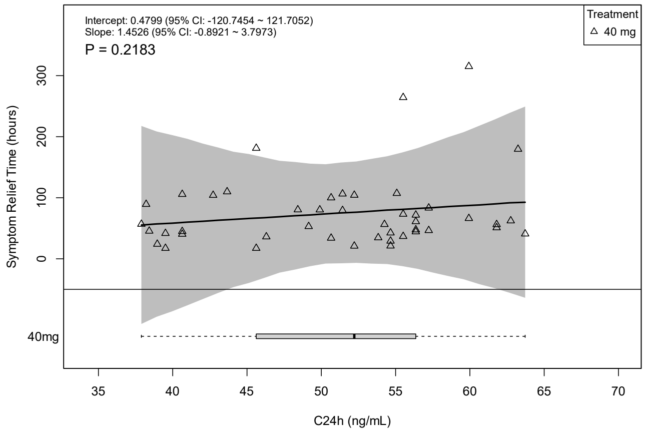

(H) adolescents: > 80kg

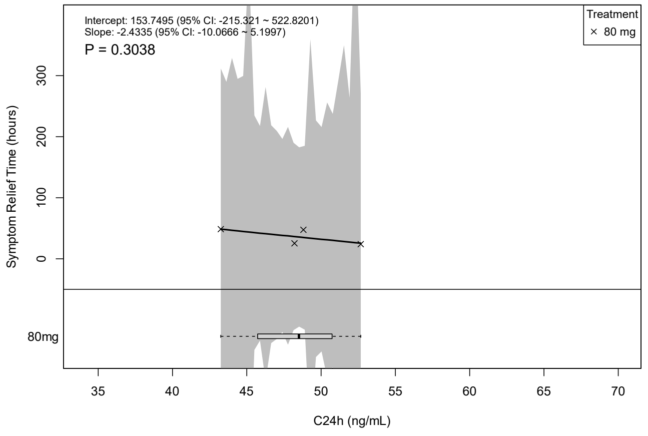

(I) adolescents: ≤ 80kg

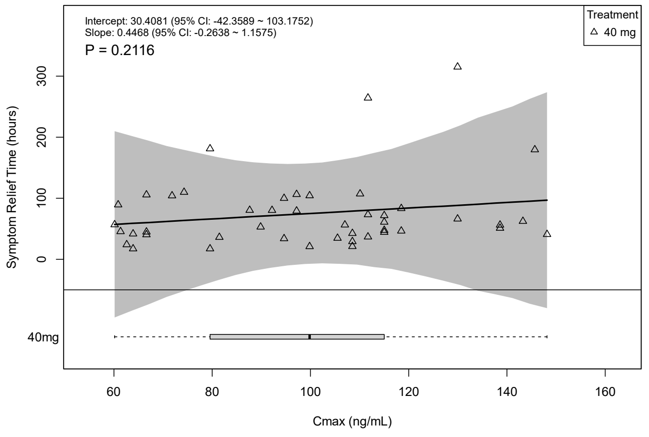

(J) adolescents: > 80kg

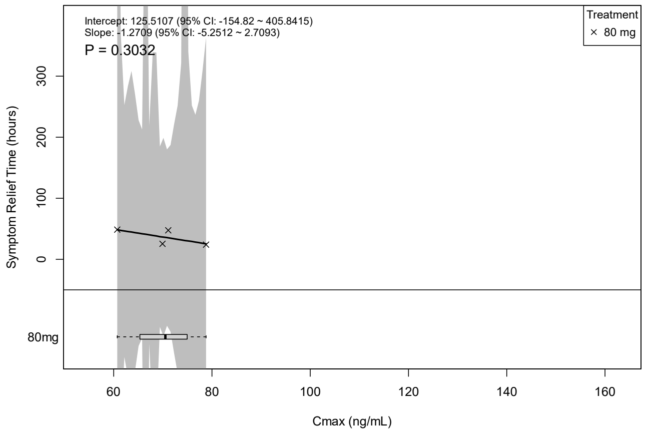

(K) adolescents: ≤ 80kg

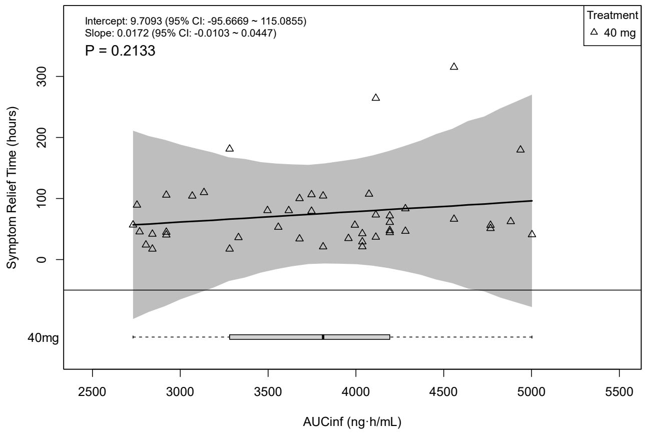

(L) adolescents: > 80kg

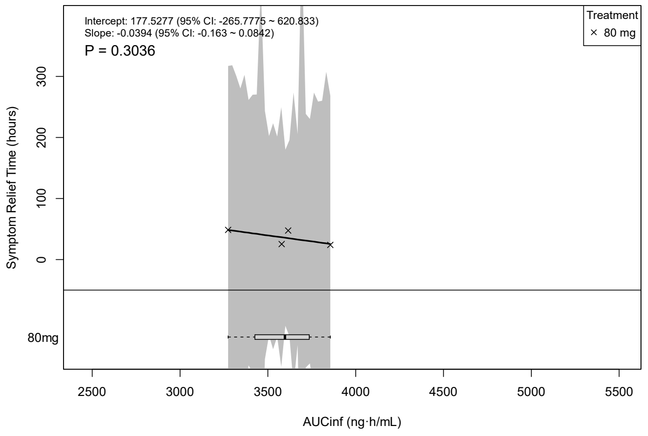

Figure S7. Sensitivity analysis of the exposure – efficacy relationship for time to alleviation of influenza-related symptoms, stratified by body weight.

For adults: exposure – efficacy relationships based on C24h (A, B), Cmax (C, D), and AUCinf (E, F), stratified by body weight (≤80 kg vs. >80 kg).

For adolescents: exposure – efficacy relationships based on C24h (G, H), Cmax (I, J), and AUCinf (K, L), stratified by body weight (≤80 kg vs. >80 kg).
