# Supplementary material for: Population Pharmacokinetics and Exposure–Response Analysis of Oral Pixavir Marboxil in Adults and Adolescents with Influenza
Source: Pharmaceutics. 2026 Apr 30;18(5):550. doi: 10.3390/pharmaceutics18050550 (PMC13210205; doi:10.3390/pharmaceutics18050550)
Supplement: Supplementary file 1 [file pharmaceutics-18-00550-s001.zip › Figure S8-flu score bs.pdf]

(A) adults: flu score ≤ 14

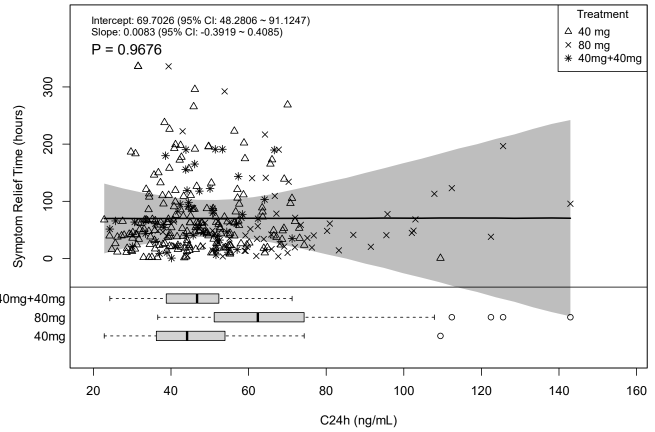

(B) adults: flu score > 14

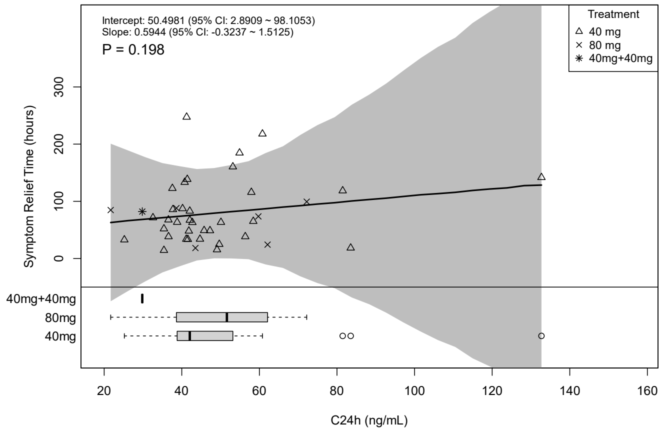

(C) adults: flu score ≤ 14

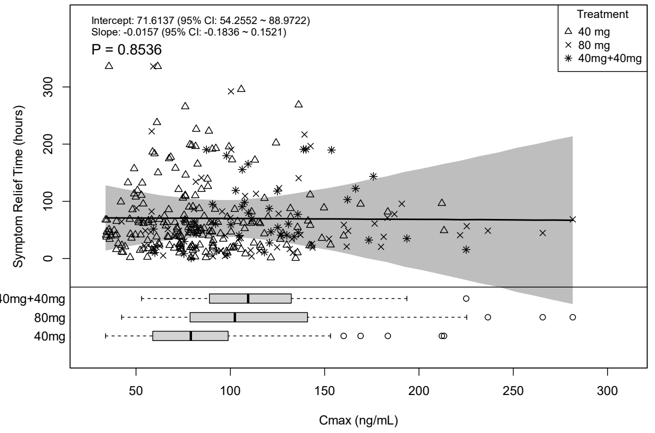

(D) adults: flu score > 14

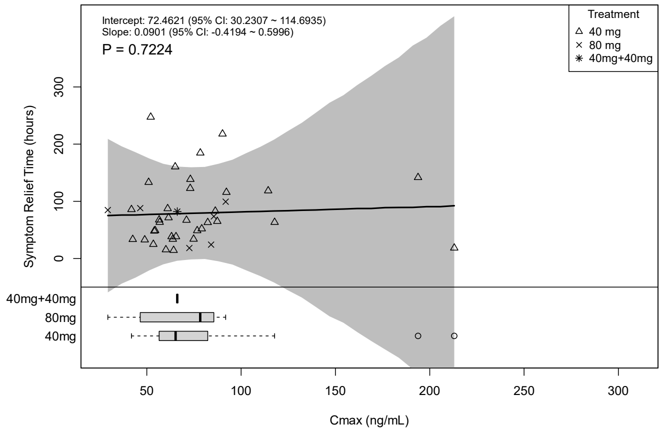

(E) adults: flu score ≤ 14

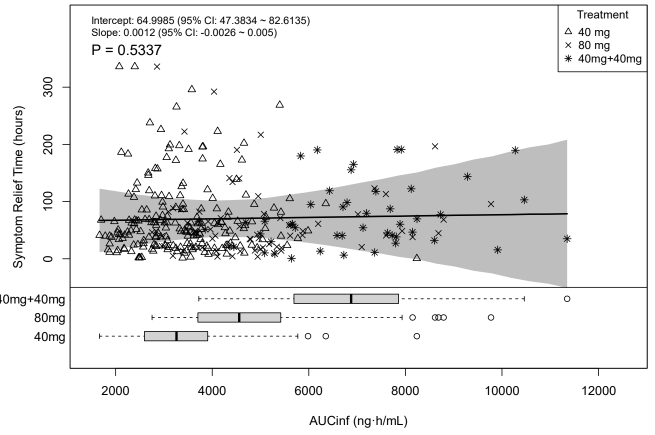

(F) adults: flu score > 14

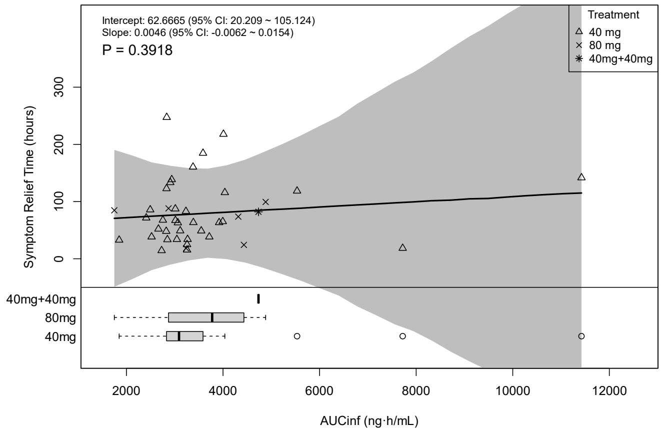

(G) adolescents: flu score ≤ 14

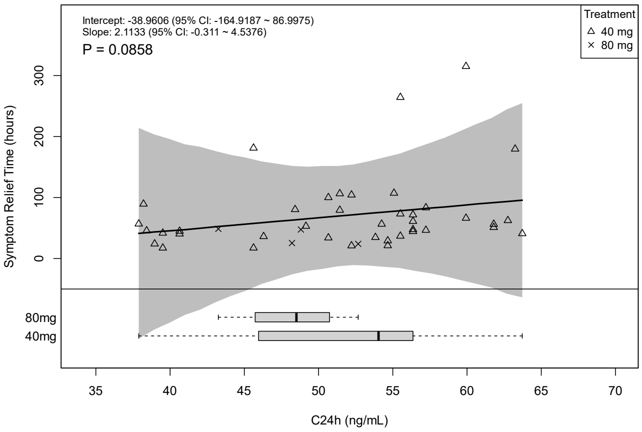

(H) adolescents: flu score > 14

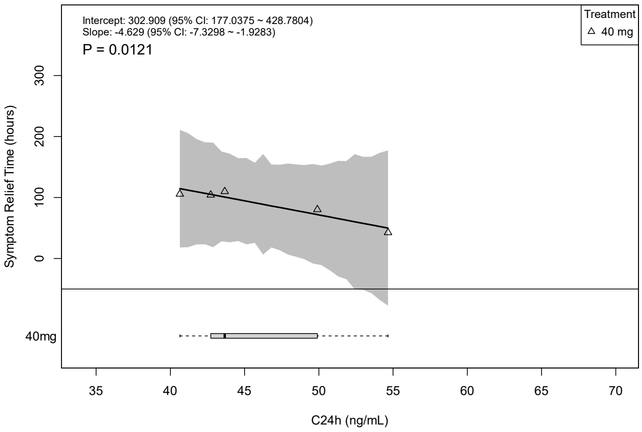

(I) adolescents: flu score ≤ 14

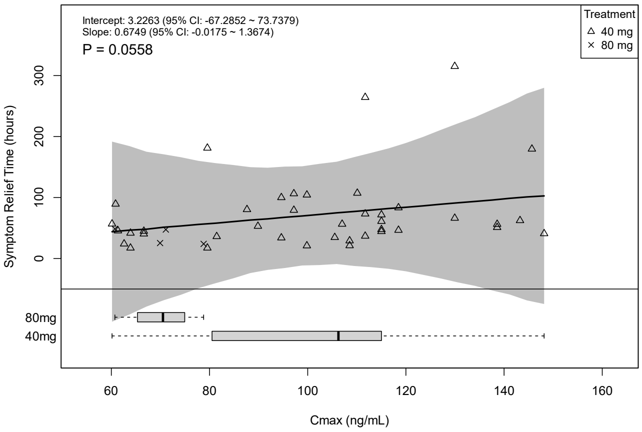

(J) adolescents: flu score > 14

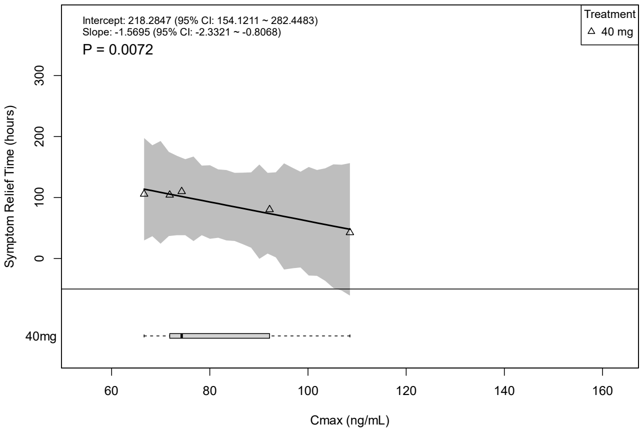

(K) adolescents: flu score ≤ 14

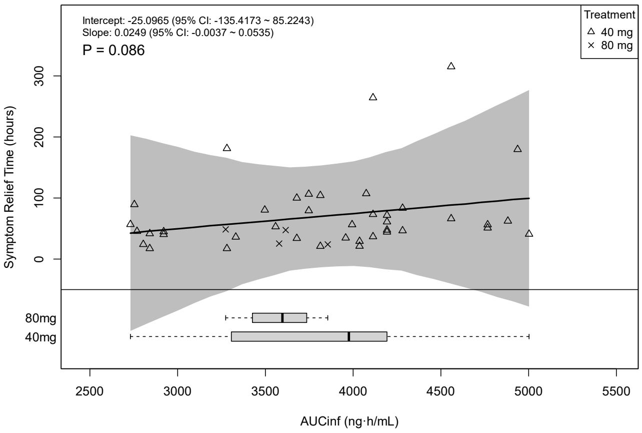

(L) adolescents: flu score > 14

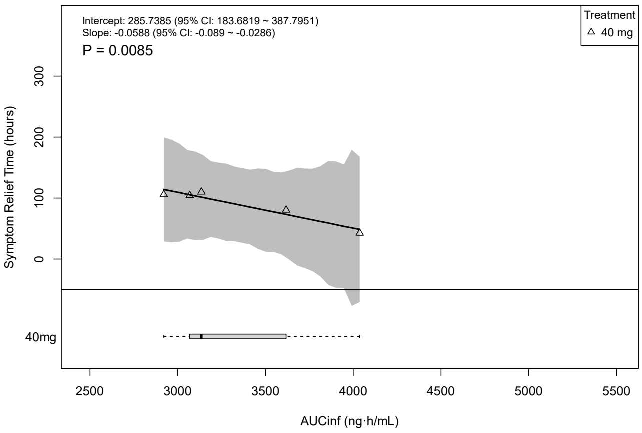

Figure S8. Sensitivity analysis of the exposure − efficacy relationship for time to alleviation of influenza-related symptoms, stratified by baseline influenza symptom score.

For adults: exposure − efficacy relationships based on C24h (A, B), Cmax (C, D), and AUCinf (E, F), stratified by baseline influenza symptom score (⩽ 14 vs. >14).

For adolescents: exposure − efficacy relationships based on C24h (G, H), Cmax (I, J), and AUCinf (K, L), stratified by baseline influenza symptom score (⩽ 14 vs. >14).
