# Supplementary material for: Population Pharmacokinetics and Exposure–Response Analysis of Oral Pixavir Marboxil in Adults and Adolescents with Influenza
Source: Pharmaceutics. 2026 Apr 30;18(5):550. doi: 10.3390/pharmaceutics18050550 (PMC13210205; doi:10.3390/pharmaceutics18050550)
Supplement: Supplementary file 1 [file pharmaceutics-18-00550-s001.zip › Label-20260202.pdf]

尺寸:380mm*600mm(上下6折,左右8折,折叠成面尺寸47*100mm)　　圣经纸:42g

壹立康®

壹立康®

核准日期:2025年12月09日
修改日期:2026年01月12日
修改日期:2026年01月22日
修改日期:2026年02月02日

玛帕西沙韦胶囊说明书

请仔细阅读说明书并在医师指导下使用

【药品名称】
通用名称:玛帕西沙韦胶囊
商品名称:壹立康®
英文名称:Pixavir Marboxil Capsules
汉语拼音:Mapaxishawei Jiaonang

【成份】
本品活性成份为玛帕西沙韦。
化学名称:[[1'-[(11*S*)-7,8-二氟-6,11-二氢二苯并[*b,e*]硫杂䓬-11-基]-1',2',4',6'-四氢-4',6'-二氧代螺[环丙烷-1,3'-[3*H*]吡啶并[1,2-*b*]哒嗪]-5'-基]氧基]甲基碳酸甲酯

化学结构式:

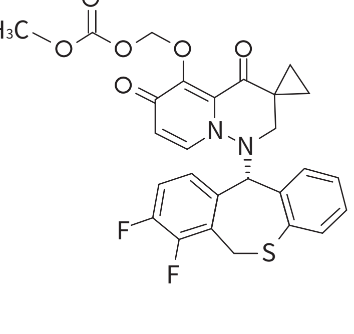

分子式:$C_{27}H_{22}F_2N_2O_6S$
分子量:540.54
辅料:聚乙烯己内酰胺-聚醋酸乙烯酯-聚乙二醇接枝共聚物、微晶纤维素、甘露醇、交联羧甲纤维素钠、硬脂酸镁、羟丙甲纤维素空心胶囊。

【性状】
本品内容物为白色粉末和颗粒。

【适应症】
本品适用于既往健康的12岁及以上青少年和成人单纯性甲型和乙型流感患者的治疗,不包括存在流感相关并发症高风险的患者。

【规格】
20mg

【用法用量】
在症状出现后48小时内单次服用本品,可与或不与食物同服。
本品适用于成人和青少年(≥12岁)单纯性甲型和乙型流感患者,基于体重的给药方案如表1所示:

表1. 基于体重的给药方案

患者体重(kg)	推荐单次口服剂量
≥40kg 至<80 kg	40 mg
≥80 kg	80 mg

剂量调整:不建议降低本品的剂量。

肾功能不全
尚未在肾功能不全患者中研究本品的安全性与有效性。

肝功能不全
尚未在肝功能不全患者中研究本品的安全性与有效性。

【不良反应】
临床试验
本品的总体安全性特征基于4项临床试验中713例接受本品治疗的受试者的数据,其中成人受试者663例,青少年受试者50例。
流感的治疗
安全性特征基于在成人和青少年单纯性流感患者中开展的1项安慰剂对照III期临床研究(TG-1000-C-03)的数据,这项研究中共500例患者接受本品治疗,包括既往健康的成人和青少年,其中450例(90.0%)为成人,50例(10.0%)为青少年(≥12岁至<18岁)。按照公斤体重,449例接受40mg剂量的本品,51例接受80mg剂量的本品。
表2列出了在该项单纯性流感III期临床试验中,接受本品治疗的成人和青少年受试者中发生率至少为1%的不良反应。

表2. 单纯性流感III期临床试验中,接受本品治疗的成人和青少年受试者中发生率至少为1%的不良反应

系统器官分类不良反应	本品(N=500)	安慰剂(N=250)
**代谢及营养类疾病**		
高尿酸血症	2.0%	2.1%
**心脏器官疾病**		
窦性心律失常	1.2%	1.3%

【禁忌】
本品禁用于已知对本品或任何辅料过敏的患者。

【注意事项】
超敏反应
本品TG-1000-C-03研究中使用玛帕西沙韦治疗的患者分别报告了1例接触性皮炎和1例荨麻疹病例,严重程度均较轻,判定均为与研究药物可能无关,无法可靠估计其发生率或确定其与本品暴露之间的因果关系。
本品禁用于已知对本品发生超敏反应的患者(参见【禁忌】)。应教育患者如何识别过敏反应症状,如果发生或疑似发生类似过敏的反应,应给予适当的治疗。

细菌性感染的风险
没有证据表明本品对除流感病毒以外其他病原体引起的疾病有效。严重细菌性感染可能以流感样症状起病,与流感并存或作为流感并发症出现。没有证据表明,本品可防止发生此类并发症。处方医师应警惕潜在的继发性细菌性感染,并在合适时给予治疗。

对驾驶和机械操作能力的影响
尚未开展对驾驶和机械操作能力影响的研究。

其他
流感病毒会随着时间发生变化,诸如病毒类型或亚型、出现耐药性或病毒毒力变化等因素可能削弱抗病毒药物的临床获益。在决定是否使用本品时,应考虑有关流行的流感病毒株药敏性的可用信息。

【孕妇及哺乳期妇女用药】
生育力
动物研究中未观察到本品对生育力有影响(参见【药理毒理】)。

妊娠期用药
尚未在妊娠女性中进行充分且对照良好的临床研究。本品对妊娠女性的潜在风险尚未可知。妊娠期内应避免使用本品,除非潜在获益大于对胎儿的潜在风险。
在动物生殖研究中,玛帕西沙韦于大鼠和家兔口服最高剂量分别为600 mg/kg和60mg/kg(其暴露量分别约为人最大推荐剂量下帕西沙韦全身暴露量的1.3倍和0.4倍),均未观察到对胚胎发育和母体的不良影响(参见【药理毒理】)。

生产和分娩
目前尚未确立本品在生产与分娩时的用药安全性。

哺乳期用药
尚不清楚本品及其活性代谢物帕西沙韦是否会分泌至人乳汁中。给予最低60mg/kg用药时,帕西沙韦被证实可以分泌至哺乳期大鼠乳汁中。因此,服用本品时需停止哺乳,服用本品至少10天后才可以恢复哺乳。应在考虑了本品对哺乳期母亲的潜在获益以及对婴儿的潜在风险后,做出停止哺乳或启动本品治疗的决定。

【儿童用药】
目前尚无12岁以下儿童使用本品的临床试验数据。确证性临床试验中包括77例12岁及以上、体重至少为40kg的青少年单纯性流感患者,详见【临床试验】。对于≥12岁的患者,用法用量参见【用法用量】。

【老年用药】
目前尚无60岁以上流感患者使用本品的临床试验数据。

【药物相互作用】
本品未开展人体药物相互作用研究。预期本品及活性代谢物帕西沙韦与细胞色素P450酶(CYP酶)底物、抑制剂或诱导剂,以及与肠道、肾脏或肝脏药物相关主要转运体的底物、抑制剂或诱导剂之间无临床显著药物-药物相互作用。

其他药物对本品的影响
体外研究显示,活性代谢物帕西沙韦不是转运体P-gp、BCRP、OATP1B1和OATP1B3的底物。

本品对其他药物的影响
体外研究显示,在临床相关浓度水平下,活性代谢物帕西沙韦对CYP3A4/5、CYP1A2、CYP2B6、CYP2C8、CYP2C9、CYP2C19和CYP2D6等亚型酶无抑制作用,对CYP1A2、CYP3A4和CYP2B6无诱导作用,对P-gp、BCRP、OATP1B1、OAT1、OAT3和OCT2的转运活性无明显抑制作用。

【药物过量】
临床经验
目前尚无有关玛帕西沙韦在人体中过量暴露的资料。根据临床试验安全性评估结果,未发现用药剂量和不良事件类型、发生率及严重程度间存在明显的相关性。由于本品临床研究未发生药物过量事件,未能确定过量用药可能引起的症状或体征。

管理
无已知的本品特效解毒剂。若发生用药过量事件,应基于患者的体征和症状启动标准支持性医疗护理。

【临床药理】
作用机制
参见【药理毒理】相关内容。

药代动力学
口服给药后,本品转化为其活性代谢物帕西沙韦。原形药物玛帕西沙韦血浆浓度低于定量检测限。

吸收
健康志愿者单次口服40mg或80mg本品后,帕西沙韦的血浆浓度达峰时间(T_{max})约为空腹给药后3.5小时。尚未确定本品的绝对生物利用度。

食物效应
健康志愿者空腹和餐后(约800~1000kcal,其中脂肪约占总热量的50%)接受本品给药的食物效应研究表明进食状态下帕西沙韦的C_{max}和AUC_{0-last}分别下降41.35%和18.76%。在进食状态下T_{max}延迟约1.50小时。在流感患者的临床研究中,本品与或不与食物同服,没有观察到临床相关的疗效差异。

分布
在体外研究中,帕西沙韦在各种属中蛋白结合率均达到96%以上。单次口服40mg本品后,流感患者中的帕西沙韦表观分布容积约为614升。

代谢
体内外研究表明,本品口服给药后,首先主要由肠道羧酸酯酶CESs催化水解代谢,生成活性代谢物帕西沙韦,再进一步经UGT1A3催化形成葡糖苷酸及发生氧化代谢。
在人体物质平衡研究中,单次口服40mg$[^{14}C]$标记本品后,帕西沙韦占总放射性血浆AUC的70.10%。也在血浆中检测出帕西沙韦的葡糖苷酸M628-2(TG-0600771,占总放射性血浆AUC的29.02%),并在尿液、粪便中检测出帕西沙韦的葡糖苷酸M628-1和帕西沙韦的亚砜产物M468-1、M468-2,确认本品通过酯水解进行体内代谢以形成帕西沙韦,随后代谢形成两种亚砜和两种葡糖苷酸。

排泄
本品和帕西沙韦在人体中主要通过粪便途径排泄。单次口服40mg$[^{14}C]$标记的本品后,粪便及尿液中排泄的总放射性物质分别占总给药放射性剂量的85.62%和5.68%。

清除
本品单次口服给药后,流感患者中帕西沙韦的表观终末消除半衰期($t_{1/2,z}$)约为36小时。

线性/非线性
空腹状态下单次口服本品后,在20~160mg剂量范围内,帕西沙韦的C_{max}、AUC_{last}和AUC_{inf}随给药剂量增加均相应增加,其中C_{max}的增加与剂量增加成正比,而AUC_{0-inf}和AUC_{0-last}的增加均小于剂量增加的比例。

特殊人群药代动力学
群体药代动力学研究显示,年龄(14-60岁)、性别不是本品显著协变量。

体重
群体药代动力学分析表明体重是一个显著协变量,随着体重增加,帕西沙韦的暴露量下降,因此,成人和儿童患者均应根据体重调整剂量。成人和青少年中,体重40kg至<80kg患者的剂量为40mg,体重≥80kg患者为80mg。当按推荐的基于体重的方案给药时,在各体重组之间未观察到暴露量存在有临床意义的差异。

肾功能不全
本品尚未在肾功能不全患者中开展临床研究。健康受试者物质平衡研究显示,本品部分经尿液排出,约占给药量的5.68%,预计肾功能不全对本品的PK特征影响较小。

肝功能不全
本品尚未在肝功能不全患者中开展临床研究。

遗传药理学
未开展相关研究。

【临床试验】
既往健康的单纯性流感的成人和青少年患者的治疗
TG-1000-C-03是一项III期、多中心、随机、双盲研究,评价单次口服本品对比安慰剂在既往健康的单纯性流感成人和青少年患者中的有效性和安全性。
在2023~2024年中国流感季,共随机入组752例发病时间≤48小时的既往健康的单纯性流感青少年和成年患者,其中青少年(≥12岁至<18岁)流感患者有77例。受试者按2:1的比例随机分配至本品治疗组和安慰剂组,共有717例接受研究药物且基线聚合酶链式反应(PCR)检测阳性的受试者,其中本品治疗组有479例,给药剂量基于体重分层,即体重≥40kg至<80kg的患者单次服用40mg本品,体重≥80kg的患者单次服用80mg本品。本研究中的主要流感病毒分别为甲型H3N2亚型(本品治疗组vs安慰剂组:87.5% vs 87.0%)和乙型(本品治疗组vs安慰剂组:10.9% vs 12.2%)。

所有流感症状缓解时间:
本研究主要疗效终点为所有流感症状(咳嗽、喉咙痛、鼻塞、头痛、发热或寒颤/发汗、疲乏、肌肉或关节痛)持续缓解时间。结果显示,发生流感的患者中,本品治疗组(479例)和安慰剂组(238例)分别为60.9小时(95%CI:52.5,63.4)和87.9小时(95%CI:84.1,96.1),Kaplan-Meier(KM)曲线图见图1。本品治疗组所有流感症状缓解中位时间较安慰剂组缩短27.0小时(95% CI:-39.0,-22.2),两组差异具有显著的统计学意义(P<0.0001)。

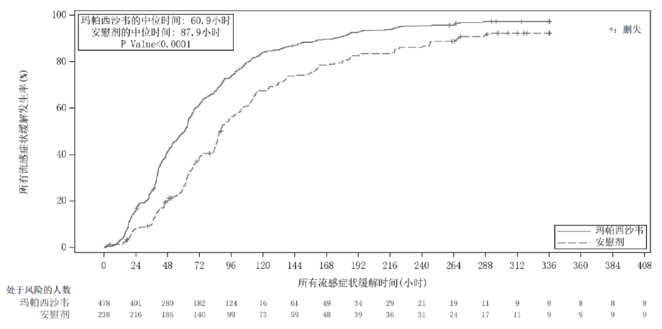

图1 所有流感症状缓解时间KM曲线图

病毒亚型
对于甲型病毒感染患者,本品治疗组(426例)和安慰剂组(209例)至流感症状缓解中位时间分别为60.9小时(95%CI:52.1,63.4)和86.6小时(95%CI:82.9,96.1)。对于乙型病毒感染患者,本品治疗组(51例)和安慰剂组(29例)至流感症状缓解中位时间分别为63.3小时(95%CI:40.4,82.9)和94.3小时(95%CI:81.9,124.4)。

青少年患者
77例≥12岁至<18岁的青少年被随机分组并接受本品(50例)或安慰剂(27例)治疗。发生流感的青少年中,本品治疗组(49例)和安慰剂组(25例)至流感症状缓解中位时间分别为53.0小时(95%CI:44.9,66.2)和114.0小时(95%CI:85.0,156.8)。
次要终点包括退热时间和基于培养评估的至病毒清除时间(通过病毒滴度测定)。

发热缓解时间:
本品治疗组的发热缓解中位时间为36.6小时(95%CI:34.8,37.9),安慰剂组为61.2小时(95%CI:54.6,64.2)。

抗病毒活性:
本品治疗组至病毒清除的中位时间(通过病毒滴度测定)为22.3小时(95% CI:21.5,22.7),安慰剂组为39.9小时(95%CI:35.2,42.5)。第3天通过逆转录聚合酶链式反应(RT-PCR)测定的流感病毒RNA较基线的变化,本品治疗组与安慰剂组差值为1.41病毒拷贝数/mL。

临床耐药性
在临床试验中,发现了甲型/H3N2流感病毒株对帕西沙韦敏感性下降的相关位点发生给药后氨基酸置换(I38T/M)。在既往健康的单纯性流感成人和青少年受试者中,根据试验TG-1000-C-02和TG-1000-C-03的汇总数据,甲型/H1N1、甲型/H3N2和乙型流感病毒感染中与帕西沙韦敏感性下降相关的给药后氨基酸置换的总发生率分别为0%(0/2)、1.2%(5/421)和0%(0/64)。青少年受试者中未发现与帕西沙韦敏感性下降相关的给药后氨基酸置换。

【药理毒理】
药理作用
作用机制
玛帕西沙韦是一种前药,通过水解转化为活性代谢产物帕西沙韦,发挥抗流感病毒活性。帕西沙韦通过抑制聚合酶酸性(PA)蛋白(病毒基因转录所需RNA聚合酶复合物中的一种流感病毒特异性酶)的核酸内切酶活性,从而抑制流感病毒复制。

抗病毒活性
在一项PA核酸内切酶试验中,帕西沙韦对甲型流感病毒(H1N1、H3N2)的50%有效浓度(EC_{50})为1.74~10.93nM,对乙型流感病毒(B/Lee/40)的EC_{50}为41.26nM。
在MDCK细胞病毒空斑减少试验中,确定了帕西沙韦抗甲型和乙型流感病毒实验室病毒株和临床分离株的抗病毒活性。帕西沙韦对甲型H1N1、甲型H3N2和乙型流感病毒的EC_{50}范围分别为0.35~1.94nM、0.91~3.09nM和2.75~11.90nM。细胞培养物中的抗病毒活性与人体临床治疗反应之间的相关性尚未确定。

耐药性
细胞培养:在持续增加帕西沙韦浓度的情况下,通过对细胞培养中病毒连续传代,选择出对帕西沙韦敏感性降低的甲型流感病毒分离株。在甲型/H1N1病毒RNA聚合酶复合物的PA蛋白中发生的氨基酸置换I38T、A36T、E199G导致甲型流感病毒对帕西沙韦的敏感性降低。

临床试验:
在临床试验中,发现了细胞培养中对帕西沙韦敏感性下降的相关位点发生给药后氨基酸置换的甲型/H3N2流感病毒株(表3)。在确诊流感病毒感染的成人和青少年受试者中,根据试验TG-1000-C-02和TG-1000-C-03的汇总数据,甲型/H1N1、甲型/H3N2和乙型流感病毒感染中与帕西沙韦敏感性下降相关的给药后氨基酸置换的总发生率分别为0%(0/2)、1.2%(5/421)和0%(0/64)。青少年受试者中未发现与帕西沙韦敏感性下降相关的给药后氨基酸置换。

表3. 与帕西沙韦敏感性下降相关的PA氨基酸置换

流感类型/亚型	甲型/H1N1	甲型/H3N2	乙型
氨基酸置换	-	I38T/M	-

交叉耐药性
由于病毒蛋白靶点不同,帕西沙韦与神经氨酸酶(NA)抑制剂或M2质子泵抑制剂(金刚烷胺类)预期不存在交叉耐药性。帕西沙韦对NA抑制剂奥司他韦耐药病毒株具有抗病毒活性,包括携带NA/H275Y突变的甲型H1N1、携带NA/E119V和NA/R293K突变的甲型H3N2病毒株。帕西沙韦对NA抑制剂扎那米韦耐药病毒株也具有抗病毒活性,包括携带NA/Q136K突变的甲型H1N1病毒株。帕西沙韦对同靶点巴洛沙韦敏感性下降的PA/I38T(A/H1N1)病毒株具有抗病毒活性。表型交叉耐药性评价的临床相关性尚未确立。

毒理研究
遗传毒性
玛帕西沙韦Ames试验结果为阳性,中国仓鼠肺成纤维细胞染色体畸变试验结果为可疑阳性,大鼠体内骨髓微核试验和彗星试验结果均为阴性,Pig-a基因突变试验结果为可疑阳性。

生殖毒性
在大鼠生育力和早期胚胎发育毒性试验中,雌性大鼠自交配前2周至妊娠第7天、雄性大鼠自交配前4周至交配期间经口给予玛帕西沙韦 60、180或600 mg/kg/天[以帕西沙韦的系统暴露量(AUC)计,约为人最大推荐剂量(MRHD)下暴露量的1.7倍],未见对生育力、交配行为或早期胚胎发育的影响。
在胚胎-胎仔发育毒性试验中,大鼠自妊娠第6天至第17天经口给予玛帕西沙韦60、180或600 mg/kg/天,兔自妊娠第6天至第19天经口给予玛帕西沙韦15、30或60 mg/kg/天,在最高剂量分别达600 mg/kg/天、60 mg/kg/天(以帕西沙韦的AUC计,约为MRHD下暴露量的1.3和0.4倍)均未见对母体和胚胎/胎仔的不良影响。
在围产期毒性试验中,大鼠自妊娠第6天至产后/哺乳期第21天经口给予玛帕西沙韦 60、180或600 mg/kg/天(以帕西沙韦的AUC计,约为MRHD下暴露量的0.9倍),所有剂量下对妊娠母体及子代均未见明显影响。帕西沙韦及代谢产物TG-0600771可通过胎盘屏障,亦可分泌至乳汁中。乳汁中帕西沙韦浓度与母体血浆中浓度相当。

致癌性
尚未开展玛帕西沙韦的致癌性研究。

【贮藏】
遮光,密封,不超过25°C保存。
请将本品放在儿童不能接触的地方。

【包装】
采用聚酰胺/铝/聚氯乙烯冷冲压成型固体药用复合硬片与药用铝箔包装,外加聚酯/铝/聚乙烯药品包装用复合膜、袋。
2粒/板,1板/袋,1袋/盒;4粒/板,1板/袋,1袋/盒。

【有效期】
24个月

【执行标准】
YBH31002025

【批准文号】
国药准字H20250070

【上市许可持有人】

名称:健康元药业集团股份有限公司
注册地址:深圳市南山区高新区北区朗山路17号健康元药业集团大厦
邮政编码:518057
电话和传真号码:0755-86252388(电话)、0755-86252020(传真)
药物警戒电话:4001821188
药物警戒邮箱:pv@joincare.com
药物警戒二维码:

网址:http://www.joincare.com/

【生产企业】

企业名称:凯莱英生命科学技术(天津)有限公司
生产地址:天津开发区第七大街71号
邮政编码:300457
电话和传真号码:022-66252888(电话)、022-66252777(传真)
网　址:https://www.asymchem.com.cn/

企业名称:健康元海滨药业有限公司
生产地址:深圳市坪山区坑梓街道金辉路11号
邮政编码:518122
电话和传真号码:0755-84531231(电话)、0755-84531231(传真)
