# Supplementary material for: Population Pharmacokinetics and Exposure–Response Analysis of Oral Pixavir Marboxil in Adults and Adolescents with Influenza
Source: Pharmaceutics. 2026 Apr 30;18(5):550. doi: 10.3390/pharmaceutics18050550 (PMC13210205; doi:10.3390/pharmaceutics18050550)
Supplement: Supplementary file 1 [file pharmaceutics-18-00550-s001.zip › Supplementary_table1.pdf]

Supplementary table 1. Comparison of noncompartmental analysis (NCA)-derived pharmacokinetic parameters from the rich-sampling Phase I cohorts with the corresponding typical estimates from the final population pharmacokinetic model.

**Parameters**	**40mg (n=20)**		**80mg (n=8)**	
	**NCA**	**PopPK**	**NCA**	**PopPK**
$AUC_{0\text{-}inf}$ (ng·h/mL)	4291.75 (21.37%)	3958.99 (19.47%)	6385.67 (18.98%)	6171.48 (17.10%)
CL/F (L/h)	9.32 (21.37%)	10.10 (19.47%)	12.53 (18.98%)	12.96 (17.10%)
Vz/F(L)	475.89 (26.79%)	414.05 (20.42%)	621.10 (23.07%)	468.22 (11.53%)
$T_{1/2}$ (h)	35.39 (12.01%)	34.28 (6.77%)	34.36 (12.38%)	31.51 (7.48%)
C_{max} (ng/mL)	143.97 (27.00%)	113.61 (27.93%)	254.88 (16.92%)	198.33 (17.02%)
T_{max} (h)	3.50 (3.00 - 5.00)	4.39 (2.41 - 7.33)	3.50 (3.00 - 6.00)	4.51 (3.42 - 6.12)

$AUC_{0\text{–}inf}$: area under the concentration–time curve from time zero to infinity, C_{max}: maximum concentration, T_{max} time to maximum concentration, T1/2 terminal elimination half–life, Vz/F apparent distribution volume, CL/F clearance Note: Data are expressed as geometric mean (coefficient of variation, CV%), except for Tmax, which is shown as median (min, max).
