# Supplementary figures and images for: Population Pharmacokinetics and Exposure–Response Analysis of Oral Pixavir Marboxil in Adults and Adolescents with Influenza
Source: Pharmaceutics. 2026 Apr 30;18(5):550. doi: 10.3390/pharmaceutics18050550 (PMC13210205; doi:10.3390/pharmaceutics18050550)

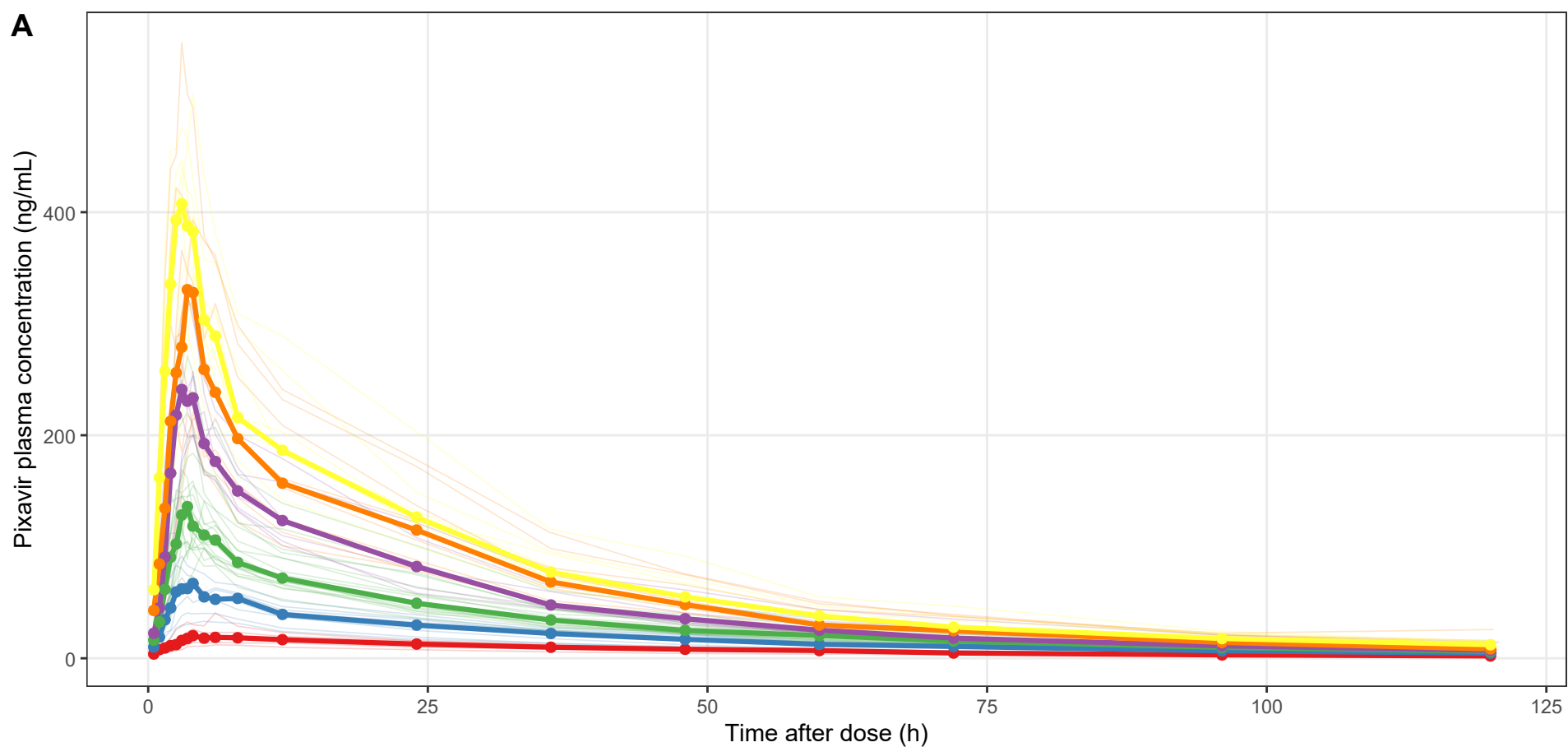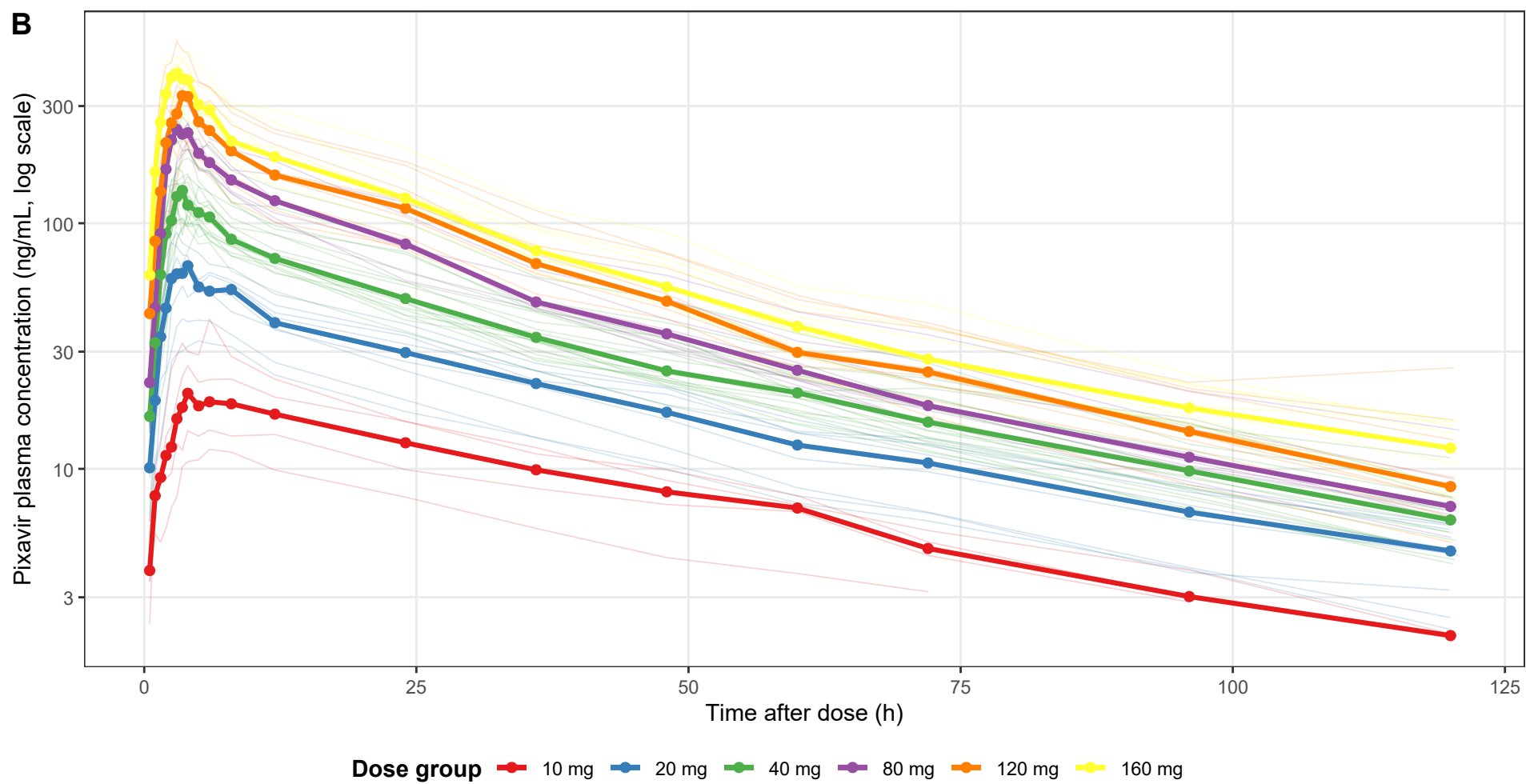

Supplement: Supplementary file 1 [file pharmaceutics-18-00550-s001.zip › Figure S1-PK profiles.pdf]

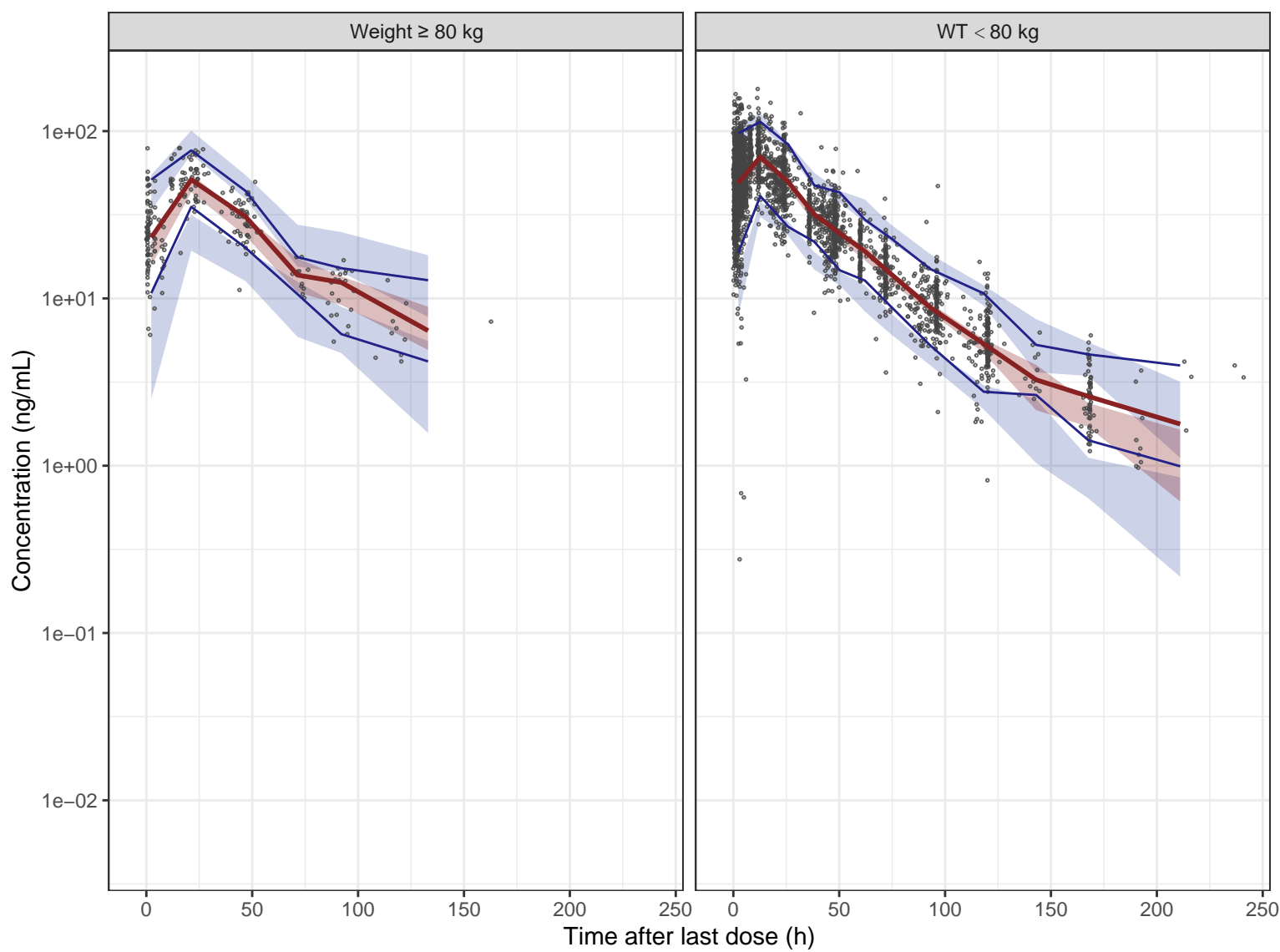

Supplement: Supplementary file 1 [file pharmaceutics-18-00550-s001.zip › Figure S2_pvcvpc_est exponent.pdf]

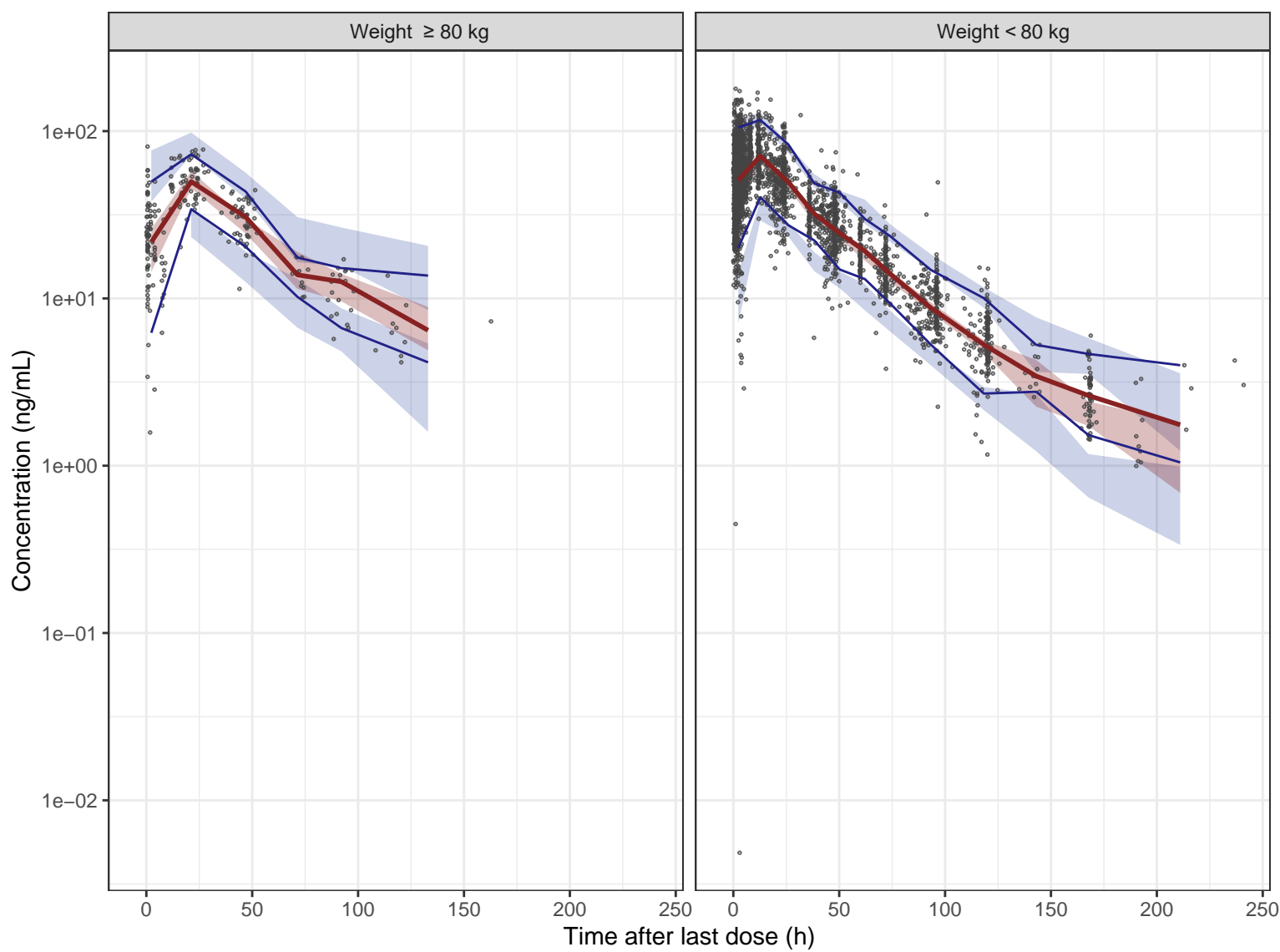

Supplement: Supplementary file 1 [file pharmaceutics-18-00550-s001.zip › Figure S3_pvcvpc_std exponent.pdf]
